# Priorities in physical therapy research: A scoping review

**DOI:** 10.1016/j.bjpt.2024.101135

**Published:** 2024-11-04

**Authors:** Sara Souto-Miranda, Eduardo Brazete Cruz, Diogo Pires, Fernando Ribeiro, Nuno Cordeiro, Cristina Jácome

**Affiliations:** a Instituto Politécnico de Setúbal, Escola Superior de Saúde, Setúbal, Portugal; b Studies and Planning Office, Portuguese Order of Physical Therapists, Lisbon, Portugal; c Comprehensive Health Research Center (CHRC), NOVA University Lisbon, Lisbon, Portugal; d Institute of Biomedicine - iBiMED, School of Health Sciences, University of Aveiro, Aveiro, Portugal; e Polytechnic Institute of Castelo Branco, Superior Health School, Castelo Branco, Portugal; f AGE.COMM-Interdisciplinary Research Unit-On Building Functional Ageing Communities, Polytechnic Institute of Castelo Branco, Castelo Branco, Portugal; g CINTESIS@RISE, MEDCIDS, Faculty of Medicine of the University of Porto, Porto, Portugal

**Keywords:** Physical therapy research, Priorities for physical therapy, Research agenda

## Abstract

**Background:**

Physical therapy is an ever-evolving profession. To improve research efficiency, it is crucial to identify knowledge gaps and establish research priorities.

**Objective:**

To review priorities for physical therapy research, and to summarize the evidence into a global research agenda. As a secondary aim we sought to compare the priorities across studies.

**Methods:**

We conducted a scoping review with searches in PubMed, Web of Science, and Google Scholar to gather studies and grey literature. Studies were included if they involved physical therapists, physical therapy researchers, patients, or policymakers who determined a research agenda or conducted a study on priority setting in physical therapy research. Content analysis was performed by two independent reviewers to gather research priorities into main topics for a global research agenda.

**Results:**

Twenty-five records were included, most being original articles (*n* = 19), from high-income countries (*n* = 25). Nine studies established generic priorities for physical therapy research, while the remaining were dedicated to physical therapy specific fields. A total of 551 priorities were established since 2000 for general physical therapy and 7 specific physical therapy areas. A global research agenda was established with 9 priority categories for future research. Cost and effectiveness studies were the more frequently prioritized research priorities.

**Conclusion:**

This review synthesized the literature on priorities for physical therapy research and provided a global physical therapy research agenda. These 9 priority categories can now be used to design future physical therapy studies and channel research efforts into questions that are relevant for multiple stakeholders and nationalities.

## Highlights

• To improve research efficiency, it is crucial to identify knowledge gaps and establish research priorities.
• This scoping review compiled all physical therapy research priorities into a global agenda for physical therapy research.
• Future physical therapy research should focus on 9 internationally applicable priorities.

## Introduction

In the most recent decades, the percentage of physical therapy research publications grew exponentially among both human health and physical rehabilitation research.^1^ For example, in recent years, cost-effectiveness studies have shown physical therapy interventions to be cost-effective in a range of conditions. Data from Australia and recent data from the United States of America, show an average net-benefit ranging from 1320 to 39,533 dollars for the management of several conditions, such as chronic obstructive pulmonary disease, carpal tunnel syndrome, and back pain.^2^, ^3^, ^4^ Development and validation of new physical therapy techniques and new methods/measures are ever emerging and are other areas responsible for this research growth.

To improve research efficiency, it is imperative to identify knowledge gaps of the profession and establish key priorities of investigation for the future. Research agendas provide clear forward-thinking viewpoints for the progression of the profession, can promote research in the field, and influence decisions of funding bodies. They also enable the alignment of research with the needs of consumers, healthcare professionals, and policy makers, reducing the research waste when there is patient and public involvement (PPI).^5^ In fact, establishing a research agenda for physical therapy, might not only reduce low quality research and channel research efforts into common and crucial goals, but also foster the development of recommendations for clinical practice, towards a contemporary, evidence and value-based physical therapy.

Although physical therapy practices, resources, and settings vary greatly around the world,^6^^,^^7^ and certain questions may be country-specific to address local policies, it is likely that most research priorities are relevant for the overall advancement of the profession. Nonetheless, to our best knowledge, physical therapy research agendas of different initiatives have never been compared nor reviewed.

Therefore, the aim of this scoping review was to identify priorities for physical therapy research, and to summarize the evidence into a global research agenda for physical therapy. As secondary aims we sought to compare the establishment of priorities across studies, in terms of the priorities chosen, the methods used, and geographical location.

## Methods

### Study design

A scoping review was conducted, as it was deemed the most appropriate method to identify how priorities have been established and to report characteristics of studies, as well as the identification of knowledge gaps.^8^ Scoping reviews have been used to summarize research priorities in other research areas.^9^ An initial search of PubMed, Web of Science, the Cochrane Database of Systematic Reviews, and JBI Evidence Synthesis was conducted, and no similar published or ongoing systematic reviews or scoping reviews were identified. The methodology of this scoping review followed the guidance of the Joanna Brigs Institute^10^ and is reported according to the Preferred Reporting Items for Systematic reviews and Meta-Analyses extension for Scoping Reviews (PRISMA-ScR) Checklist.^11^ The protocol of the scoping review was registered in the Open Science Framework (https://osf.io/whs4m).

### Eligibility criteria

Using the Population, Concept, and Context (PCC) framework advised by the Joanna Brigs Institute,^10^ we searched for documents with physical therapists, physical therapy researchers, patients, or policy makers who determined a research agenda (i.e., a set of priorities for future research) in any region of the world or any sub-area of physical therapy (Table 1).

**Table 1:** Population, Concept, and Context (PPC) framework of the scoping review.

**Population**	Physical therapists, physical therapy researchers, patients, or policy makers
**Concept**	A research agenda (i.e., a set of priorities for future research) or study on priority setting in physical therapy research
**Context**	Any region of the world or any sub-area of physical therapy

Documents with established research priorities for physical therapy through stakeholder perspectives, including those of physical therapists, patients, researchers, and policy makers, were included. In this work stakeholder is defined as a person/group of persons “with an interest or concern in something, especially a business. Denoting a type of organization or system in which all the members or participants are seen as having an interest in its success.”^12^ Qualitative work, such as interviews, focus groups, surveys, meetings, as well as research articles or other documents for practice and policy, including policy statements, clinical guidelines, and editorials, were eligible for inclusion. Studies were eligible if published from 2000 onwards. This timeline was chosen as physical therapy research has substantially grown since that year^13^ and we aimed to provide a list of contemporary and time-appropriate research priorities. Additionally, the reference lists of all included records were screened to identify any relevant additional documents. Conversely, studies that did not meet these criteria, were abstracts, quantitative research designs, commentaries, or literature reviews. No language restrictions were imposed.

### Search strategy, source of evidence screening, and selection

A comprehensive electronic search was conducted in March 2023 to locate both published and unpublished documents. Search alerts were set to update the review until publication. A search of PubMed and Web of Science was undertaken to identify studies on the topic. Unpublished studies/grey literature were searched in Google Scholar.

The search strategy for PubMed included: ("research priorit*"[Title/Abstract] OR "research agenda"[Title/Abstract] OR "priorit* setting"[Title/Abstract] OR "priorit* research"[Title/Abstract] OR "agenda setting"[Title/Abstract]) AND ("physiotherapy"[Title/Abstract] OR "physical therapy"[Title/Abstract]). The strategy was adapted for each included database (S1).

Following the search, all identified citations were uploaded into EndNote 20 (Clarivate Analytics, PA, USA) and duplicates removed. Titles and abstracts were screened by two independent reviewers for assessment against the inclusion criteria for the review. Authors were contacted up to three times when the full text of an article was not available.

The full text of selected citations was assessed in detail considering the inclusion criteria. Reasons for exclusion at the stage of full text review were recorded. Any disagreements between reviewers were solved through consensus with additional team members.

### Data extraction

Data were extracted from documents into a pre-developed data extraction table by one author (S. S-M) and verified by another independent reviewer (CJ). The data extracted included specific details: authors’ name, year of publication, country, study design (e.g., interviews, Delphi survey), physical therapy field (i.e., musculoskeletal, neurological, pediatric, cardiorespiratory, pre and post-operative, oncology, and wellbeing), stakeholders involved (i.e., patients, clinicians, researchers, policy makers), data collection procedures, and the list of priorities of each study. Disagreements were solved with a third team member (EB.C).

### Analysis and presentation of results

A content analysis was employed to map research priorities and create a global research agenda.^14^ Priorities were first coded and then merged by similar semantic meanings. A summative content analysis approach was used by two independent researchers, who coded the information into categories until consensus was reached, which could be achieved with the input of the additional team members.^14^ Summative content analysis is a method that quantifies content to better understand its contextual use and explore usage, with latent content analysis (interpretation of the underlying meanings of words).^14^ The number of research priorities within each category was used to rank research categories. Data are presented in a descriptive summary of the main findings and are charted and tabulated in a detailed manner.

## Results

### Study selection

**Fig. 1:**
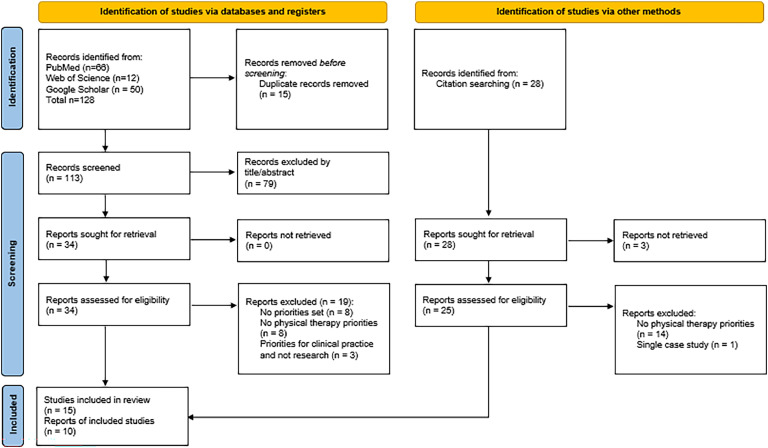
Flow diagram of included studies (*n* = 25).

A total of 128 records were retrieved from database searches, and an additional 28 were found through citation searching. Of the 59 full-text records assessed for eligibility, 22 did not report physical therapy-specific priorities, 8 did not set any priority, 3 had priorities established for clinical practice rather than research, and 1 was a case study. Hence, 25 documents were included in this review.^15^, ^16^, ^17^, ^18^, ^19^, ^20^, ^21^, ^22^, ^23^, ^24^, ^25^, ^26^, ^27^, ^28^, ^29^, ^30^, ^31^, ^32^, ^33^, ^34^, ^35^, ^36^, ^37^, ^38^, ^39^ The results of the search and the study inclusion process are reported in the PRISMA flow diagram (Fig. 1).

### Characteristics of studies

Of the included documents, 19 were original articles,^15^, ^16^, ^17^, ^18^, ^19^, ^20^^,^^22^, ^23^, ^24^, ^25^, ^26^, ^27^, ^28^, ^29^^,^^31^^,^^33^^,^^36^^,^^38^^,^^39^ 5 institutional reports,^30^^,^^32^^,^^34^^,^^35^^,^^37^ and 1 a Masters thesis.^21^ In terms of geographical distribution, all were from high-income countries. Fourteen were conducted in Europe,^16^^,^^17^^,^^19^^,^^21^^,^^23^, ^24^, ^25^, ^26^^,^^32^^,^^34^, ^35^, ^36^^,^^38^^,^^39^ 9 in North America,^15^^,^^20^^,^^22^^,^^28^, ^29^, ^30^, ^31^^,^^33^^,^^37^ and 2 in Asia.^18^^,^^27^ Country details can be found in Table 2.

**Table 2:** Characteristics of included studies (*n* = 25).

Author, year	Country	Study design	Physical therapy field(s)	Stakeholders involved	Data collection	Number of priorities established for physical therapy
Beattie P. et al., 200,0^15^	USA	Series of conference meetings and survey	Generic	All members of the Section on Research of APTA All academic administrators of physical therapist education programs (APTA members) All component presidents A random sample of clinical specialists certified by the ABPTS A random sample of members of APTA 4 consultants and 3 APTA staff members to review questions *N* = 227 respondents Sex: NR Age: NR	2 conferences to identify clinical research questions answerable in 5 years. **Phase 1:** First conference to gather research questions. Between each conference consultants and staff members edited questions. **Phase 2:** Incorporation of other questions from consultants on health services research for the second meeting. **Phase 3:** Respondents rated each question based on the importance of the question to a clinician and how often the clinician would use the answer to a particular question in clinical practice. Score based on the 2 questions on a 5-point Likert scale - a sum of the average importance rating and the average frequency rating. **Phase 4:** 4 levels of priority were used: level 1 contained those questions for which 40 % or more of the respondents rated the question as extremely clinically important and occurring very often in clinical practice; Level 3 contained those questions for which 10 % or more of the respondents rated the questions as unimportant or occurring infrequently; the boundaries of level 2 were scores for questions that fell between the criteria for level 1 and level 3; the fourth level included questions that had score values of <6.6 Subgroup analysis compared responses of members of the research section with nonmembers.	72
Soma et al., 200,9^27^	Japan	3-round Delphi survey	Generic	Physical therapists (*n* = 46) 85 % male 30–69 years old	**Round 1:** List 3 high priority research questions (43 % response rate) **Round 2:** Rate on a 5-point Likert scale (1-not that important to 5-extremely important) 53 research questions (63 % response rate) **Round 3:** Feedback and new ratings (52 % response rate) **Consensus:** Research questions <4 quartiles of the top 25 % were excluded	13
Rushton & Moore, 201,0^26^	UK	Modified Delphi survey	Musculoskeletal	Tutors of students (*n* = 39) and clinical experts/physical therapists (*n* = 52) nominated by member organizations of the IFOMPT Sex: NR Age: NR	**Round 1:** >10 priorities for postgraduate dissertation/theses (68 % response rate) Content analysis to identify themes/subthemes **Round 2:** Rate with a 5-point Likert scale 23 themes and sub themes identified, add suggestions (67 % response rate) **Round 3:** Feedback and new ratings (58 % response rate) **Consensus:** Mean rating ≥3.5, coefficient of variation ≤30 %	43
McDonough, 201,1^35^	Ireland	3-round Delphi survey	Generic	Physical therapy experts (physical therapists employed at clinical and academic settings with ≥3yrs experience) *N* = 34 23.5 % male 25–65 years old	**Round 1**: Qualitative (What do you think are the research priorities for your profession at present?) **Round 2:** Survey with items from Round 1 answered with a 5-point Likert scale from “most important” to “least important” **Round 3:** Feedback on Round 2 with possibility of changing scores **Consensus:** a70 % agreement Mean scores used to rank priorities	21
Goldstein et al., 201,1^20^	USA	Discussions within APTA sections experts and consultants	Generic	APTA sections experts Basic scientists Physical therapists Health services researchers N: NR Sex: NR Age: NR	**Phase 1:** Content generation by APTA sections **Phase 2:** A 6-person Consultant Group was appointed by the APTA Board of Directors and asked to complete 2 tasks: review feedback (items) submitted from the sections and, devise a conceptual framework to organize items that would be consistent and minimize redundancy **Phase 3:** The draft of the Research Agenda, as created by the Consultant Group, then was sent to each of the Sections for a final review	80
Rankin et al., 201,2^24^	UK	Modified 3-round Delphi survey	Musculoskeletal physical therapy Neurology physical therapy Cardiorespiratory physical therapy Wellbeing	1 expert panel for each of the 4 fields of physical therapy addressed musculoskeletal *n* = 61, neurology *n* = 60, cardiorespiratory *n* = 43, and Wellbeing *n* = 40 Experts in clinical practice (*n* = 125), research (*n* = 94), education (*n* = 87), management/service provision (*n* = 34), service commissioning/planning/purchasing(*n* = 8), policymaking (*n* = 22), guideline panel membership (*n* = 28), and users of physical therapy services, charities, and patient organisations (*n* = 19) Sex: NR Age: NR	**Round 1**: Request up to 5 priorities and supporting statements for research topics for physical therapy in the UK **Round 2:** Feedback in the form of the themes (thematic analysis of round 1) grouping the research topics with supporting statements. Rating of topics using a 1–5 Likert scale, higher scores higher importance **Consensus**: Mean rating ≥3.5, coefficient of variation ≤30 %, ≥55 % agreement, Kendall's coefficient of concordance **Round 3:** Feedback and new ratings of consensual items from round 2	127
Pollock et al., 201,2^38^	UK	Mixed-methods study with 4 phases	Neurology- life after stroke	• Stroke patient groups (*n* = 15) • Stroke individual survivors (*n* = 22) • Caregivers (*n* = 4) • Health professional groups (*n* = 4) • Health professionals (*n* = 61) Prioritisation by 3 independent groups of: • Health professionals (*n* = 55) • Stroke survivors/caregivers (*n* = 42) • Sex: NR • Age: NR	**Phase 1:** Gathering of treatment uncertainties through postal and electronic surveys, visits to stroke groups and professional meetings, and evidence searches and analysis **Phase 2:** Checking of evidence (systematic reviews/guidelines) for the 548 uncertainties submitted, 226 unanswered **Phase 3:** Interim prioritisation through surveys, visits to stroke survivors and professional meetings. Ranking the 10 most important priorities from the list. Resulted in 25 research priorities **Phase 4**: Consensus meeting to achieve consensus on the top 10 priorities. Ranking of priorities in order of importance combined to give a total score.	1
Gierisch et al., 201,4^33^	USA	Evidence synthesis and online surveys	Musculoskeletal (Osteoarthritis)	*N* = 13 Clinical experts Researchers Representatives of funding agencies Professional societies Consumer/patient advocacy group (Arthritis Foundation) Policy makers	**Phase 1:** Evidence synthesis from systematic reviews, clinical practice guidelines, and documents on research needs identified 31 evidence gaps. **Phase 2:** 13 stakeholders provided input on the evidence gaps. Forced-ranking prioritization method on online survey	1
Boney et al., 201,5^16^	UK	2-round survey and prioritisation workshop	Pre and postoperative physical therapy	Healthcare professionals (*n* = 388) Patients (*n* = 304) Caregivers/friends (*n* = 299) Patient organisations (*n* = 3) Anaesthetic professional societies (*n* = 8) Sex: NR 98 % between 25 and 75 years old	**Phase 1:** Online survey asked to submit up to three ideas for research **Phase 2:** Thematic analysis to classify suggestions Literature reviews to explore if questions had been answered and to search for additional ones **Phase 3:** Interim prioritisation where participants were asked to select the 10 most important research questions **Phase 4:** Exclusion of any questions nominated by >90 % of respondents only from the group of clinicians or only lay respondents **Phase 5:** Prioritisation from most to least popular (least frequently chosen to be on the top 10) **Phase 6:** Final workshop with a modified Delphi process – group discussions and ratings	1
Morris et al., 201,5^36^	UK	Surveys, evidence searches, workshops	Neurology	Paediatricians (*n* = 44) Paediatric neurologists (*n* = 7) Surgeons (*n* = 12) Nurses (*n* = 12) Speech and language therapists (*n* = 29) Physical therapists (*n* = 61) Occupational therapists (*n* = 39) Orthotist/prosthetists (*n* = 4) Psychiatrists (*n* = 6) Psychologists (*n* = 1) Dentists (*n* = 1) Teachers (*n* = 2) Academic/researchers (*n* = 2) Specialist health visitor (*n* = 1) Audiologist (*n* = 1) Orthotist (*n* = 1) Administrator/manager (*n* = 1) General practitioners (*n* = 2) Health professionals (not specified, *n* = 11) Parents and health professionals (*n* = 5) Part of an organisation supporting disabled people (*n* = 29) Parents, carers, relatives (*n* = 183) Young persons with a neurodisability (*n* = 11) Persons with neurodisability ≥25 years old (*n* = 8) Sex: NR Age: NR	**Phase 1:** Design survey to gather uncertainties from families and clinicians **Phase 2:** Survey was pilot tested and refined **Phase 3:** Additional research recommendations were extracted from relevant guidelines Aggregation of questions by type of impairment/diagnosis and by intervention Systematic reviews to verify if research questions have been answered **Phase 4:** Interim prioritisation where the top 10 preferences were ranked Question with rank 1 = 10 points, rank 2 = 9 points; rank points went down such that rank 10 = 1 point Steering group agreed on 25 top questions **Phase 5:** Workshop face-to-face with a modified nominal group technique – participants were asked to rank the 25 questions, and these were discussed until agreement	2
Nast et al., 201,6^23^	Switzerland	Focus groups and individual interviews, 2-round Delphi survey	Generic	Physical therapy researchers (*n* = 38) Physical therapy practitioners (*n* = 199) Physical therapy educators (*n* = 41) Representatives of patient organisations (*n* = 26) Representatives of health organisations (*n* = 9) Representatives of health insurers (*n* = 5) Physicians (*n* = 18) Nurses (*n* = 27) Occupational therapists (*n* = 17) Physical educators (*n* = 9) Other health professionals (*n* = 9) Health politicians (*n* = 19) Sex: NR Age: NR	**Phase 1:** Focus group discussions (*n* = 18) and semi-structured interviews (*n* = 23) to identify research areas **Round 1 of Delphi:** Prioritisation of questions with ranking from 1 to 10 and agreement with statements from 1 to 5 **Round 2:** Final consensus process Ranking of priorities from 1 “highest importance” to 10 “lowest importance” **Consensus:** High if C ≤ 1.00, moderate if C = 1.01–2.00, or minor if C = 2.01–3.00. C=interquartile range/4	21
Rangan et al., 201,6^39^	UK	Online survey, online and face-to-face meetings	Musculoskeletal	Steering Committee composed of patients, physical therapists, general practitioners, shoulder surgeons, anaesthetists, orthopaedic nurses, academic clinicians, James Lind partnership coordinator, data analyst Participants of survey: patients, caregivers, and clinicians (*n* = 371) Sex: NR Age: NR	**Phase 1:** Initial awareness meeting with stakeholders **Phase 2:** Request to participants to identify uncertainties **Phase 3:** Questions and uncertainties refined by data analyst **Phase 4:** Interim prioritisation through online survey and Steering Committee meeting to reduce list of priorities – responses of “yes”, “no”, or “unsure” **Phase 5:** Face-to-face meeting with group discussions and plenary sessions. Groups rotated until there was agreement over the top 10 uncertainties	2
KNGF, 201,7^34^	The Netherlands	Development of a knowledge framework in meetings, online survey	Neurology Cardiorespiratory Musculoskeletal Oncology	*N* = 69 Scientists Research institutes Healthcare professionals Policy makers Health and professional organisations Sex: NR Age: NR	**Phase 1:** Framework developed by the KNGF and physical therapy professors **Phase 2:** Survey requesting participants to supply a top 3 of knowledge gaps in the form of short, SMART (Specific, Measurable, Acceptable, Realistic, Time-bound) described, research questions **Phase 3:** Review of questions against evidence in Cochrane and other systematic reviews **Phase 4:** Review of other national agendas and discussion with other stakeholders **Phase 5:** Questions categorised into themes **Phase 6:** Testing of agenda to see if it was in line with a national policy document **Phase 7:** Final input from the stakeholders on the physical therapy science day **Phase 8:** Final version agreed by the board of directors of KNGF	12
CSP, 201,8^32^ and Rankin et al., 202,0^25^	UK	Online survey, literature reviews, and prioritisation workshop	Generic	Patients (*n* = 174) Caregivers (*n* = 44) Physical therapists working in clinical practice (*n* = 234) Physical therapy support workers (*n* = 6) Physical therapy students (*n* = 13) Physical therapy researchers (*n* = 69) Physical therapy educators (*n* = 40) Physical therapy managers (*n* = 26) Other healthcare professionals (*n* = 30) Others (*n* = 74) 22 % male Mean age = 47 years old	**Phase 1:** First initial awareness meeting to promote the priority setting partnership **Phase 2:** Online survey to identify uncertainties and evidence searches (searches for 2 themes by a qualitative researcher: Developing and sharing models of good practice for reducing the burden on secondary care; and promoting good practice in primary care for people with multiple morbidities **Phase 3:** Thematic analysis and verification of priorities answered in systematic reviews **Phase 4:** Interim prioritisation through an online survey **Phase 5:** Final prioritisation workshop – final ranking of the 25 questions, including top 10 Ranking of priorities by highest total score Equal weight to both groups (patients and physical therapists)	25
Gomes et al., 201,8^21^	Portugal	3-Round Delphi survey	Musculoskeletal	Clinical experts (*n* = 27) Educators (*n* = 22) Clinical educators (*n* = 29) Masters in musculoskeletal physical therapy (*n* = 9) Patients (*n* = 28) 46 % male 28–60 years old	**Round 1:** Request identification of 3–5 research priorities Content analysis to identify themes **Round 2 and 3:** Scoring items on a 5-point Likert scale from 1 “not important” to 5 “extremely important” **Consensus**: mean rating≥4, median≥4, coefficient of variation ≤30 %, ≥80 % agreement, Kendall's coefficient of concordance (W)	10
Fernandez et al., 201,8^19^	UK	Paper and online survey, workshop	Musculoskeletal	Steering group consisted of patient representatives, healthcare professionals and carers with established links to relevant partner organisations Participants (*n* = 365) were healthcare professionals (51 %), family and friends (23 %), patients (16 %), and caregivers (10 %) Sex: NR Age: NR	**Phase 1:** National scoping survey asking respondents to submit their research uncertainties **Phase 2:** Uncertainties from respondents compiled with other from relevant national guidelines published by the National Institute for Health and Care Excellence **Phase 3:** Thematic analysis to define themes of research questions **Phase 4:** Evidence searches to ensure uncertainties were not already answered **Phase 5:** Pilot test of the survey Interim prioritisation survey to rank importance of each indicative question on a 5-point Likert scale, from 1 “not important” to 5 “extremely important” Questions ranked by mean score **Phase 6:** Final workshop with discussions to achieve consensus on the top 10 from the 25 research questions	3
Wilson et al., 201,9^29^	Canada	Online survey, evidence searches	Neurology	Physical therapists (*n* = 59) 85 % male Age 20–69 years old	**Phase 1:** Questionnaire informed by authors’ clinical and theoretical knowledge, available literature and practice recommendations **Phase 2:** Draft reviewed by 11 physical therapists and researchers who were experts on the subject 5-point Likert scale from “never” to “very often” Participants could provide additional items	6
Moerchen et al., 202,0^22^	USA	4-round Delphi survey	Education in paediatric physical therapy	Physical therapists with academic leadership roles (*n* = 11), from a summit (*n* = 12), academy members (*n* = 12), non-paediatric physical therapy educators (*n* = 12), and clinical and residency educators (*n* = 10) Sex: NR Age: NR	**Round 1 of Delphi survey:** Creation of initial set of categories of research questions Content and thematic analysis **Round 2:** Refinement of data collected in round 1 and list of the most important topics **Round 3:** Consensus from 21 statements based on agree/strongly agree scorings **Consensus**: ≥80 % among all participants or 3 or more stakeholder groups **Round 4:** Prioritisation of items through average rank scores	12
APTA, 202,1^30^	USA	Environmental scan, conceptual framework and survey	Generic	Scientific and Practice Affairs Committee APTA component presidents Practice chairs Research chairs APTA council leaders The Foundation for Physical Therapy Research Board of Trustees The Federation of State Boards of Physical Therapy N: NR Sex: NR Age: NR	**Phase 1:** Stakeholders identified the top 3 research categories from a list of 6. **Phase 2:** Based on the survey results, Population Health Research, Clinical Research and Health Services Research were selected as the priority categories **Phase 3:** From the compiled research agenda and based on frequency, themes, and importance to the profession the Scientific and Practice Affairs Committee selected 3 research items in each of the 3 selected categories for a total of 9 priorities	9
APTA Pediatrics Section 202,1^37^ and Bhat et al., 202,2^31^	USA	Video-conferencing meetings and survey	Paediatric physical therapy	Task force of 7 people from the APTA Pediatrics Section First feedback from: expert physical therapist, APTA Pediatrics Director of Practice, APTA Pediatrics Practice Knowledge Translation Committee Chair, Pediatric Physical Therapy journal editor-in-chief, and APTA Pediatrics Research Grants Committee Chair Second feedback from: APTA Pediatrics leadership, clinicians, research team members, parents and families of children with special needs, and self-advocates	**Phase 1:** Inclusion of new topics based on feedback of previous agenda **Phase 2:** Monthly meetings to discuss “next great idea” as possible research topics. Report from the meetings was used as base to revise the APTA Pediatrics agenda **Phase 3:** Review of goals and research agendas of different institutions and alignment of the agenda with those entities **Phase 4:** Inclusion of priorities related to research in COVID-19 and diversity, equity, and inclusion´ **Phase 5:** Feedback from experts (8 respondents) **Phase 6**: Public comment period of the revised agenda with feedback from several stakeholders who responded to a survey with the questions: 1) Does this research agenda cover the major research areas important for studying health conditions of infants, children, and adults? 2) Do the appendices provide sufficient examples of the different research areas? 3) Are there any major research areas missing? 4) What other examples should we be adding to the research areas? The first 2 questions were rated on a Likert scale from “not at all” = 1 to “most definitely” = 7. **Phase 7:** Analysis of median scores. Comments for questions 3 and 4 informed future drafts **Phase 8:** Incorporation of feedback and publication on the institutional websites.	62
VanSwearingen et al., 202,2^28^	USA	Adaptation of the APTA Research Agenda	Generic	Physical therapists/researchers/policy makers Sex: NR Age: NR	**Phase 1:** Adaptation of the APTA Research Agenda by authors **Phase 2:** Approved by the Academy of Geriatric Physical Therapy Board of Directors	25
Bowring et al., 202,2^17^	UK, Luxemburg, Germany	Survey	Neurology	People with Parkinson's (*n* = 511), 62 % male Healthcare professionals (total *n* = 112) Others (family members, carers (*n* = 77)	**Phase 1:** Scoring of top 10 on a Likert scale **Phase 2:** Questions were ordered by the percentage agreeing that it was a high priority (score 7–9) **Phase 3:** Subgroup comparisons with Cohen's kappa Added questions by participants were thematically analysed	1
Dijkstra et al., 202,3^18^	Qatar	2-round international Dephi survey and 2 online meetings	Musculoskeletal	Steering Committee composed of physicians (*n* = 4), allied healthcare professionals (*n* = 6), and health researchers (*n* = 3) Orthopaedic surgeons (*n* = 11), patients and public (*n* = 10), physical therapists (*n* = 17), physicians (*n* = 13), radiologists (*n* = 6), researchers (*n* = 8) 60 % male Age: NR	**Round 1:** Items from literature and steering committee's knowledge, scored on a Likert scale from 1 (not important) to 9 (critical), with option of new items proposed by participants **Round 2:** Feedback by stakeholder group and rescoring of items Thematic analysis of individual and group feedback; Dissent analysis (bipolarity of group opinion, outlier analysis, and stakeholder group analysis) **Consensus in:** very important (7–9) by ≥70 % of panel members and not important (1–3) by <15 % of panel members **Consensus out:** scored as not important (1–3) by ≥70 % of panel members and very important (7–9) by <15 % of panel members Meetings with interactive group processes with discussion of results Final ranking with the Essential National Health Research ranking strategy - score on a 3-point Likert scale regarding appropriateness, relevancy, chances of success, and impact of the research outcome	3

ABPTS, American Board of Physical Therapy Specialties; APTA, American Physical Therapy Association; CSP, Chartered Society of Physical therapy; IFOMPT, International Federation of Orthopaedic Manipulative Physical Therapists; NR, not reported; OMT, orthopaedic manipulative therapy; PT, physical therapy.

The majority of studies (*n* = 15) employed more than one methodologic approach to define the research priorities.^16^^,^^18^^,^^19^^,^^23^^,^^25^^,^^29^, ^30^, ^31^, ^32^, ^33^, ^34^^,^^36^, ^37^, ^38^, ^39^ Most studies (*n* = 23) conducted surveys.^15^, ^16^, ^17^, ^18^, ^19^^,^^21^, ^22^, ^23^, ^24^, ^25^, ^26^, ^27^^,^^29^, ^30^, ^31^, ^32^, ^33^, ^34^, ^35^, ^36^, ^37^, ^38^, ^39^ Nine studies used the Delphi method.^16^^,^^18^^,^^21^, ^22^, ^23^, ^24^^,^^26^^,^^27^^,^^35^ Fourteen studies used expert meetings,^15^^,^^16^^,^^18^, ^19^, ^20^^,^^25^^,^^28^^,^^32^^,^^34^^,^^36^, ^37^, ^38^, ^39^ and 9 conducted evidence searches to explore if research priorities have been already answered, or to gather priorities from other sources.^16^^,^^19^^,^^25^^,^^29^^,^^32^, ^33^, ^34^^,^^36^^,^^38^ One study conducted focus groups and individual interviews.^23^

Most studies described the sample included (*n* = 22),^15^, ^16^, ^17^, ^18^, ^19^^,^^21^, ^22^, ^23^, ^24^, ^25^, ^26^, ^27^^,^^29^^,^^31^, ^32^, ^33^, ^34^, ^35^, ^36^, ^37^, ^38^, ^39^ and an average of 286 stakeholders participated (min=13, max=1002). The most frequently involved stakeholder groups were physical therapists (*n* = 20 studies),^15^^,^^18^^,^^20^, ^21^, ^22^, ^23^, ^24^, ^25^, ^26^, ^27^, ^28^, ^29^, ^30^, ^31^, ^32^^,^^34^, ^35^, ^36^, ^37^, ^38^ researchers and other academics (*n* = 16 studies),^15^^,^^18^^,^^20^^,^^22^^,^^23^^,^^25^^,^^26^^,^^28^^,^^30^, ^31^, ^32^, ^33^, ^34^, ^35^, ^36^, ^37^ and patients (*n* = 14 studies).^16^, ^17^, ^18^, ^19^^,^^21^^,^^23^, ^24^, ^25^^,^^31^^,^^32^^,^^36^, ^37^, ^38^, ^39^ Seven studies^17^^,^^23^, ^24^, ^25^^,^^32^^,^^36^^,^^38^ identified differences between stakeholder group ratings, such as practitioners and educators giving a higher priority for the development of the profession,^23^ representatives of health insurers, organizations, and occupational therapists giving higher priority for research topics around physical therapy in multidisciplinary networks^23^; funding conditions being more important to physical therapists than patients^32^; and communication being more important for family members than for healthcare professionals.^36^

Nine of the 25 studies established generic priorities for physical therapy research,^15^^,^^20^^,^^23^^,^^25^^,^^27^^,^^28^^,^^30^^,^^32^^,^^35^ while the remaining were dedicated to physical therapy specific fields, namely: musculoskeletal (*n* = 8),^18^^,^^19^^,^^21^^,^^24^^,^^26^^,^^33^^,^^34^^,^^39^ neurological (*n* = 6),^17^^,^^24^^,^^29^^,^^34^^,^^36^^,^^38^ pediatric (*n* = 3).^22^^,^^31^^,^^37^ cardiorespiratory (*n* = 2),^24^^,^^34^ pre and post-operative (*n* = 1),^16^ oncological (*n* = 1),^34^ and wellbeing (*n* = 1).^24^

**Fig. 2:**
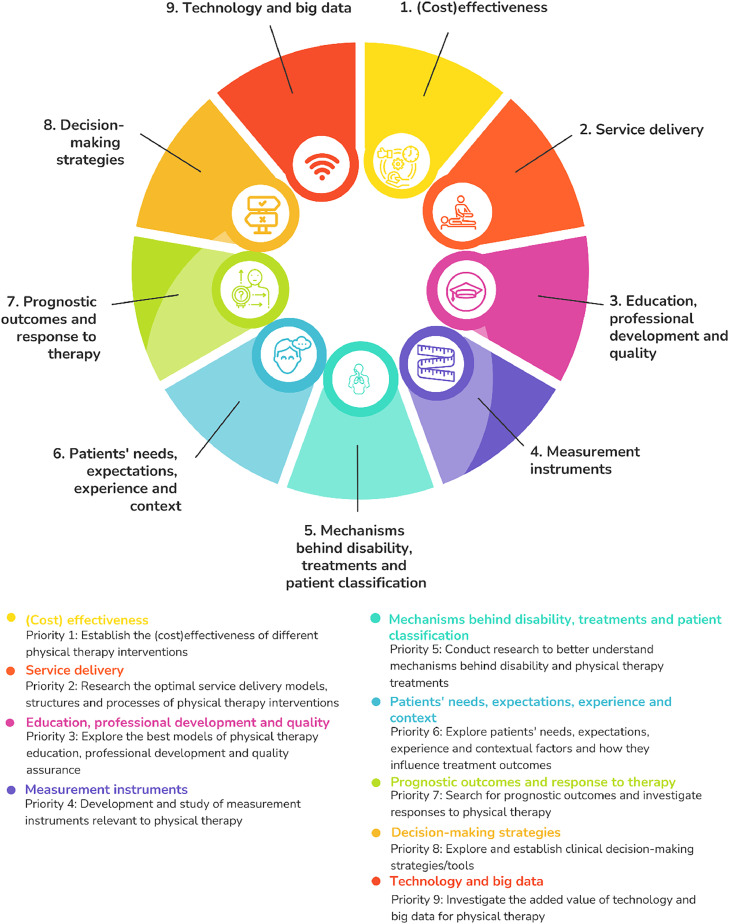
Global research agenda for physical therapy with 9 research priorities resultant from content analysis of 25 documents with research priority setting for physical therapy.

A total of 551 priorities were established between 2000 and 2023. Following the content analysis, 9 research priority categories were identified: 1) establish the (cost)effectiveness of physical therapy interventions (202 research questions), 2) research the optimal service delivery models, structures, and processes of physical therapy interventions (*n* = 86), 3) explore the best models of physical therapy education, and professional development and quality (*n* = 63), 4) develop and study measurement instruments relevant to physical therapy (*n* = 56), 5) conduct research to better understand mechanisms behind disability, physical therapy treatments, and patient classification systems (*n* = 52), 6) explore patients' needs, expectations, experience, and contextual factors and how they influence treatment outcomes (*n* = 42), 7) search for prognostic outcomes and investigate responses to physical therapy (*n* = 27), 8) explore and establish clinical decision-making strategies/tools (*n* = 21), and 9) investigate the added value of technology and big data for physical therapy (*n* = 20). The global physical therapy research agenda can be visualized in Fig. 2. Full characteristics of included studies and the list of research priorities within each priority of the global agenda can be found in Table 2 and Supplementary online material (S3), respectively. Table 3 summarizes the research priority categories.

**Table 3:** Summary of the 9 research priorities.

Research priority category	Examples of key-questions
(Cost)-effectiveness	• Are manual techniques effective in the treatment of impairments and functional limitations? • Cost benefit analysis of the provision of services • Contrasting the clinical effectiveness of the use of classes with one-to-one treatment approaches • The impact of exercise intensity on symptom management and recovery in long term conditions • Evaluate the comparative cost and/or cost-effectiveness of specific physical therapy interventions compared with or in combination with other interventions.
Service delivery	• Are there optimal time periods for interventions that influence pathology, impairment, functional limitation, and disability in patients in whom multiple episodes of care are expected over the life span? • What is the optimal resource schedule and utilization to achieve a desired effect or outcome for a given diagnosis? • How have changes resulting from health care reorganization affected the quality of physical therapy services, access to physical therapy services, patient satisfaction, staff productivity, staff longevity, and professional development? • How does the requirement of referral before treatment affect whether patients have access to and are likely to utilize physical therapy services? • An exploration of the impact of the pressure of targets, waiting lists, and the volume of repeat referrals on achieving intervention outcomes that reflect the needs and expectations of patients
Education, professional development, and quality	• Is the physical therapy practiced based on evidence? • What approaches in education assist in the development of clinical reasoning skills? • Determine the best methods to foster career development and leadership in physical therapy • Evaluate the effect of clinical education models on clinical outcomes, passing rates on the National Physical Therapy Examination, and employment settings after graduation • Evaluate methods to enhance adherence to recommended practice guidelines
Measurement instruments	• What are the psychometric properties of performance-based and self-assessment measures of physical function designed to predict functional limitations and disability in elderly people? • What is the predictive value of a broad range of assessment tools? • How is patient satisfaction evaluated? • Develop new tools or refine existing tools to measure the impact of physical therapy on activity, participation, and quality of life • Which core outcome sets of patient-relevant and crucial (generic if possible) outcome measures and minimally clinically relevant improvements should be used by physical therapists in daily practice?
Mechanisms behind patient disability, treatments, and patient classification	• What is the best method of motion learning? • Investigate the factors that modify the response to physical therapy intervention and positive tissue adaptation (e.g., genetic, functional, structural, psychosocial, and physiological factors) • Determine the mechanisms by which physical therapy interventions modify disease and age-related or injury-induced changes in normal cellular structure and function using appropriate human and animal models • What are the physiological effects of different physical therapy treatments? • Develop and evaluate effective patient/client classification methods to optimize clinical decision making for physical therapist management of patients/clients.
Patients’ needs, expectations, experience, and context	• Do patient knowledge, attitude, culture, understanding, and expectations affect the outcome of physical therapy interventions, and, if so, how? • What are the factors that motivate patients to adhere to a plan of care? • How does the physical environment in which the patient must function (eg, work requirements, mobility barriers) influence the effectiveness of treatment interventions? • Do payer source and policies influence satisfaction with access to physical therapy services in patients with acute conditions? • What factors contribute to patient satisfaction?
Prognostic outcomes and response to therapy	• What tests and measures should be used to predict the physical therapy services patients will require upon discharge from inpatient care to achieve maximum function? • What impairment-level and functional-level measures predict work capacities? • What factors appear to predict outcome of care in individual subgroups? • Identify factors that predict the risks of, or protection from, health conditions (injury, disorders, and disease) • What factors predict the onset of health problems, patient responses to physical therapy, or their abilities to make health changes/self-manage? Which patients (if any) are likely to benefit most/least from physical therapy?
Decision-making strategies	• What information from the diagnosis/prognosis is used in patient/client management? • What factors are used by physical therapists to determine their recommendations of settings to which patients are discharged? • Develop and test the effectiveness of decision support tools to facilitate evidence-based physical therapist decision making • Evaluate the effect of physical therapist post professional specialty training on clinical decision making and patient/client outcomes • Evaluate the effectiveness of shared clinical decision-making schemes between the patient/client and therapist on clinical outcomes and costs
Technology and big data	• Identify technologies to assist physical therapists in developing prevention approaches that optimize outcomes • What is the feasibility and added value of ‘internet-based care’ or ‘blended care’, aiming at enhancing patient adherence and sustained treatment effects, compared to completely supervised physical therapy, usual care, or no intervention (‘wait-and-see-policy’)? • What is the possible role of ‘big data’, collected through technological devices, in monitoring health (reductions) and physical functioning in specific patient groups, or in identifying diseases in an early phase in healthy people? • Investigate the effects of technology on the effectiveness of physical therapy interventions, participation, and quality of life (e.g. robotic devices, wearable technologies, interactive gaming systems, virtual reality systems, adaptive exercise equipment, digital health, telehealth, and mobile health) • Create aggregated, harmonized datasets from multiple ongoing studies and/or legacy data from past research studies using common data elements and share data with other researchers for further secondary analysis.

## Discussion

This review synthesized the literature on priorities for physical therapy research and compiled research priorities into a global physical therapy research agenda. These 9 priority categories can now be used to design future physical therapy studies and channel research efforts into questions that are relevant for multiple stakeholders (e.g., physical therapists, patients, regulatory authorities) and nationalities.

Defining the cost-effectiveness of physical therapy interventions and the best structure and processes of the interventions were two of the areas with more priorities established. An Australian study has documented the cost-effectiveness of physical therapy for 11 conditions.^2^ However, there are global disparities in service delivery and resources among countries,^6^^,^^7^ and therefore conducting an economic evaluation might be necessary for a vast number of conditions in different countries, to demonstrate to the public, policy makers, and insurance companies the added value of physical therapy within the healthcare landscape. Additionally, defining the optimal service delivery models, especially the structure of interventions (e.g., setting, resources – human and material, knowledge) and processes (e.g., waiting lists, timing of treatment, components of interventions, referral rates), together with defining the core outcomes of interventions, can aid quality assurance.^40^^,^^41^ This can be achieved through an iterative process of assessing these indicators and making adjustments to practice, commonly performed under the framework Plan-Do-Study-Act (PDSA).^42^

The third category with highest number of research priorities was education, professional development, and quality. Education in physical therapy and professional development (pursuit of short courses, master and doctoral degrees) varies greatly globally. Nevertheless, to protect citizens, it is imperative to ensure that physical therapy degrees have a minimum quality and that physical therapists evolve as new evidence and techniques arise. Researchers should investigate new education models and compare them with more traditional models (e.g., problem-based learning/flipped classroom vs. standard theoretical and practical lectures),^43^^,^^44^ and the added-value of short or long-term courses/degrees on the skillset of the physical therapist. In the interim, educators and professionals can use the physical therapist education framework developed by the World Physiotherapy, to develop the physical therapy curricula to minimum standards, and advance the physical therapist from novice to expert, keeping in mind the specificities and needs of each physical therapy specialty.^45^

Regarding measurement instruments, although developing instruments relevant for physical therapy is important, studying their psychometric properties and feasibility in clinical practice (e.g., associated cost, space needed, training required) is equally if not more valuable, to avoid using measures of poor quality and low applicability. In fact, systematic reviews of measurement properties, commonly expose a lack of clinimetric data in original studies.^46^^,^^47^ Additionally, similar to other fields, it is possible that physical therapists misuse measurement instruments despite their limitations.^48^ Hence, future reviews of measurement instruments using the COnsensus-based Standards for the selection of health Measurement Instruments (COSMIN) methodology could be conducted,^49^ to determine issues with physical therapy instruments and ascertain needs of future research in this field.

Another key priority for physical therapy research is to better understand the mechanisms behind disability and classification systems, and especially why some treatments might work or not. Physical therapy is an ever-evolving area with new techniques being frequently implemented in clinical practice before their effectiveness is well established or the rationale for their use clearly understood.^50^ Hence, researchers should prioritize investigating the mechanisms that explain the effects of different therapies. The Rehabilitation Treatment Specification System (RTSS), a theoretical framework developed for this purpose, can be used to guide the design and reporting of studies.^51^

Patients’ needs, expectations, experience, and contextual factors can contribute to the clinical reasoning of the physical therapist and the success of an intervention.^52^^,^^53^ Hence, it is important to study physical therapy interventions from the patient's point of view, to improve the health alliance between the patient and the physical therapist, and optimize treatment outcomes. Additionally, having PPI in research, and using patient-reported outcome measures (PROMs) and patient-reported experience measures (PREMs) might foster the adoption of a truly patient-centered physical therapy approach.^54^^,^^55^

Although it was not a highly ranked category, investigating clear prognostic criteria, patients’ classification, and understanding how patients might respond to a certain therapy is important to aid clinical decision making. Predicting patient's disability based on cut-offs of measurement instruments can help tailor physical therapy treatments,^56^ and therefore diagnostic test studies are encouraged. Similarly, responder analyses have been conducted in hopes to understand why some patients do not achieve a clinically meaningful outcome with physical therapy, and to try to estimate *a priori* if they will be responders or not to an intervention, to ultimately choose the best clinical paths for each patient.^57^^,^^58^ Furthermore, researching and establishing the best decision-making strategies is key. In fact, tools such as decision trees, and artificial intelligence might be useful for clinical practice, with a greater research investment in this area needed.^59^, ^60^, ^61^

The last category for physical therapy research identified was to investigate the added value of technology and big data for physical therapy. In the last years, a vast amount of technology-aided physical therapy interventions has rose. These can go from simple wearable technology to inform physical activity interventions, to a full virtual therapist for home-based physical therapy.^62^ Nonetheless studies showing the reliability of these mHealth tools and their added value to standard clinical practice are scarce. With the crescendo of commercial devices, it is imperative to consistently conduct research in this area. Moreover, although the use of big data in physical therapy is only marginal, analyzing big datasets from electronic health records can inform strategies for continuous improvement of health services, and should therefore be a priority for the future.^63^

In this review we found that the research methodology of included studies varied greatly, with less than half of studies using the Delphi methodology. This is consistent with the many methods described as useful to achieve a list of priorities for research,^64^ and with a systematic review conducted for research priority setting for Black and minority ethnic health.^65^ This however contrasts with a review for dementia, where up to 70 % of studies had a Delphi or multi-step design.^66^ Our review also found patient participation in 56 % of studies, which is below the engagement in other fields (65–70 % of studies),^65^^,^^66^ and few participations of policy makers (24 % of studies). These results highlight the need to establish the optimal study design for establishing research priorities, and the promotion of PPI initiatives, facilitating the involvement of non-experts, such as patients, carers, decision makers, and citizens.

Except for 1 study conducted in 3 different countries, all other research priority setting exercises of the included documents were developed for specific regions. Thus, future studies investigating research priorities could combine views from different countries, to establish internationally applicable research agendas. Furthermore, although the research priorities in the original studies cover a range of physical therapy areas, to our knowledge there is no priority setting document for women's health or geriatrics. It is possible that some physical therapy-related priorities of these areas are embedded in priority setting documents of other health-areas and could have been missed by our search strategy. Yet, it is still important to understand from the clinicians, patients, and researchers’ point of view, which gaps of the literature should be filled in these areas.

This review provides a global agenda for physical therapy research which can be useful for physical therapy researchers designing new studies. Nonetheless, some limitations need to be acknowledged. Our search was restricted to priorities relevant for physical therapy, and therefore other priorities that could be relevant for the profession might have been excluded (e.g., generic priorities for rehabilitation). Searching for priorities applicable to physical therapy in any field of medicine would be dependent on our judgement and could increase the level of bias. Moreover, we did not conduct searches to verify if research questions have already been answered in the literature, and therefore this step should be performed in the future. Our search was conducted in English, which might have hindered our ability to identify important reports of non-English speaking countries. Most research questions identified were developed in North America, Europe, and Asia, and therefore their applicability for low- and middle-income countries, and specifically the South American, African, and Australian continents is unclear. Hence, future research should explore the applicability of these research questions for such regions or develop new ones. Our scoping review was designed and conducted without PPI, which could have provided an important viewpoint for the methodology and findings. Nevertheless, using the global physical therapy research agenda gathered, a priority setting exercise can be conducted grounded in PPI, to ascertain the research priorities in each country/region. Finally, we did not find priorities related to some emergent topics such as exoskeleton-assisted therapy, or physical therapy during emergency situations (e.g., natural disasters, pandemics), which will likely be important topics in the future.

## Conclusions

This review provides a global agenda for physical therapy research, with 9 research priority categories that should be explored. Researchers can use this research agenda to confirm the relevance of these priorities in their context/regions (e.g., low- and middle-income countries), to design studies, and conduct relevant and contemporary investigations to answer these questions.

## Declaration of competing interest

The authors declare no competing interest.

## Appendix Supplementary materials

Image, application 1
