# Supplementary material for: Priorities in physical therapy research: A scoping review
Source: Braz J Phys Ther. 2024 Nov 4;28(6):101135. doi: 10.1016/j.bjpt.2024.101135 (PMC11570230; doi:10.1016/j.bjpt.2024.101135)
Supplement: Supplementary file 1 [file mmc1.pdf]

Supplementary online material

S1. Search strategy

Search strategy for Web of Science

TI=(research prior* OR research agenda OR priorit* setting OR priorit* research OR agenda setting) AND TI=(physiotherapy OR physical therapy)

Search strategy for Google Scholar

allintitle: "research priorit*" OR OR OR "research agenda" OR OR OR "priorit* setting" OR OR OR "priorit* research" OR " OR agenda OR setting" OR AND OR "physiotherapy" OR OR OR "physical therapy")

Note: The search was conducted using the following string on the advanced search "at least one word" in the article title, and date from 2000: ("research priorit*" OR "research agenda"OR "priorit* setting"OR "priorit* research" OR "agenda setting") AND ("physiotherapy" OR "physical therapy")

S2. All priorities established for physical therapy research

Table S2. Priorities established for each study (n=25).

Author, year	Priorities
Beattie P. et al., 2000[1]	1. What factors can be used to classify patients with thoracic disorders?<br>2. What factors can be used to classify patients following a cerebrovascular accident?<br>3. Do motor control strategies differ in people with low back pain compared with people without low back pain, and, if so, how?<br>4. What are the relationships between self-report of function and observed measures?<br>5. What are the psychometric properties of performance-based and self-assessment measures of physical function designed to predict functional limitations and disability in elderly people?<br>6. What is the reliability of segmental mobility testing in the cervical spine?<br>7. What are the reliability and validity of assessment of pronation of the foot in patients with knee pain?<br>8. What is the reliability of the McKenzie classification system for the cervical spine?<br>9. What information from the diagnosis/prognosis is used in patient/client management?<br>10. What factors beyond the diagnosis/prognosis determine patient/client management?<br>11. When multiple tests and measures are used, how is the information weighted in determining a diagnosis?<br>12. What combination of examination data can be used to guide clinical decision making for patients with pain in the sacroiliac region?<br>13. How does information from the systems review influence tests and measures chosen?<br>14. How does information from the history influence tests and measures chosen?<br>15. Do measures of postural alignment in people with spinal disorders influence clinical decision making, and, if so, how?<br>16. What factors influence the transfer of functional skills from the therapeutic environment to the community?<br>17. What are commonly performed physical functional tasks, and how do they differ across the life span?<br>18. Do measures of impairment and function predict a person's ability to work or return to work?<br>19. What are the variables, if any, that predict return of function in individuals following stroke?<br>20. What impairment-level and functional-level measures predict work capacities?<br>21. What information from measures can be used to predict physical function in community-dwelling elderly people?<br>22. What measurements of ambulation are useful for predicting patients' function?<br>23. Are there measures that can be used to predict independent function in an urban community, and, if so, what measures and at what thresholds?<br>24. Are there measures of ambulation that can be used to predict independent function in various communities, and, if so, at what thresholds?<br>25. Are there elements of motor control and cognitive function that can be used to predict physical function in individuals with central nervous system dysfunction?<br>26. Are there measurements from the initial examination that predict future or concurrent mobility or disability, and, if so, how?<br>27. What factors are used by physical therapists to determine their recommendations of settings to which patients are discharged?<br>28. What tests and measures should be used to predict the physical therapy services patients will require upon discharge from inpatient care to achieve maximum function?<br>29. What are the modifiable risk factors for cumulative trauma syndrome?<br>30. How are responses to exercise different in patients with neurological impairments?<br>31. How do impairments affect disability in patients?<br>32. To what extent do variables such as pharmacology, psychosocial factors, and environmental factors influence the relationship among impairment, functional limitation, and disability in people receiving physical therapy interventions?<br>33. Are there critical levels and elements of motor control that must be present to permit household ambulation in individuals with brain dysfunction, and, if so, what are they?<br>34. What are the characteristics of people who respond to various forms of therapy for low back pain?<br>35. Do patient knowledge, attitude, culture, understanding, and expectations affect the outcome of physical therapy interventions, and, if so, how?<br>36. How can patient characteristics and environmental factors be used to predict adherence to home programs?<br>37. What are the factors that motivate patients to adhere to a plan of care?<br>38. Is there a relationship between a patient's satisfaction with care and adherence to his or her physical therapy care plan?<br>39. How does the physical environment in which the patient must function (eg, work requirements, mobility barriers) influence the effectiveness of treatment interventions?<br>40. How does the environment in which the patient must function influence the choice of physical therapy interventions?<br>41. What is the effectiveness of segmental mobilization/manipulation in reducing impairment and improving functional outcomes in patients with reduced segmental mobility?<br>42. Are manual techniques effective in the treatment of impairments and functional limitations?

	43. What is the effect of exercise (duration, intensity, and type) on bone density?
	44. Can physical therapy interventions for patients with spasticity or rigidity improve function?
	45. What interventions designed to change movement strategies can be used for patients with lumbar segmental instability, and what is the optimal pattern?
	46. What interventions designed to decrease pain and paresthesias can be used for patients with upper-extremity entrapment syndromes, and what is the optimal pattern?
	47. What is the effect of various intensities and durations of intervention on the rate and degree of functional recovery after anterior cruciate ligament injury?
	48. Is there a relationship between weight-bearing exercises and the risk of fractures for people with bone demineralization, and, if so, what is the relationship between exercise and risk?
	49. What are the conditions of repetition and practice (whole/part, intermittent/continuous, attended/unattended, number of trials per day) that optimize function in people with neuromuscular dysfunction?
	50. Are there optimal time periods for interventions that influence pathology, impairment, functional limitation, and disability in patients in whom multiple episodes of care are expected over the life span?
	51. What is the optimal dose/response relationship for interventions (eg, aerobic and strengthening exercise, manual therapy, physical agents, traction/mechanical modalities, flexibility), given a specific category of a classification system for low back pain?
	52. Are there changes to behavior and the environment that can be used to enhance function and prevent impairments, and, if so, what is the optimal pattern of use to achieve a therapeutic outcome?
	53. Does immediate postoperative physical therapy intervention improve the rate of recovery of function in patients with impaired cardiovascular function, and, if so, how?
	54. Does immediate postoperative physical therapy intervention affect the rate of recovery of function in patients following orthopedic surgery, and, if so, how?
	55. Can interval training be used to improve physiological and functional outcomes in frail elderly people? If yes, can the process of interval training be standardized with frail elderly people?
	56. Are outcomes of treatment following peripheral nerve injury using neuromuscular re-education improved by early assessment and staged interventions?
	57. What is the relative effectiveness of immobilization versus mobilization in patients with musculoskeletal impairments on tissue healing and recovery of function?
	58. Does the coordination of exercise and surgical interventions affect patient outcomes, and, if so, what is the optimal pattern of intervention?
	59. Does the coordination of exercise and pharmacological interventions affect patient outcomes, and, if so, what is the optimal pattern of intervention?
	60. What are the interactions between physical therapy interventions and pharmacological interventions?
	61. What is the optimal resource schedule and utilization to achieve a desired effect or outcome for a given diagnosis?
	62. What are the factors that affect cost for physical therapy services within specific diagnostic groups?
	63. Which, if any, devices and equipment (assistive, adaptive, orthotic, protective, supportive, or prosthetic) can be used by physical therapists to enhance function and prevent impairments, and what is the pattern of use to achieve a therapeutic outcome?
	64. Do changes to behavior and the environment reduce the incidence of work-related cumulative trauma disorder?
	65. Do physical therapists' knowledge, attitude, culture, understanding, and expectations affect the outcome of physical therapy interventions, and, if so, how?
	66. Is there a difference in patient outcomes and costs dependent on whether services for a given diagnostic condition are provided by physical therapists or others?
	67. How have changes resulting from health care reorganization affected the quality of physical therapy services, access to physical therapy services, patient satisfaction, staff productivity, staff longevity, and professional development?
	68. Do payer source and policies influence satisfaction with access to physical therapy services in patients with acute conditions?
	69. Do payer source and policies influence satisfaction with access to physical therapy services in patients with chronic conditions?
	70. How does the requirement of referral before treatment affect whether patients have access to and are likely to utilize physical therapy services?
	71. What is the effect of the availability, cost, and payment source of physical therapy services on patient outcomes?
	72. What are the factors that determine whether patients have access to and are likely to utilize physical therapy services?
Soma et al., 2009[2]	1. Is the difference between natural recovery and the effect of the treatment clear?
	2. For how long and for what sort of patient should the physical therapy be done?
	3. What are the necessary criteria for motion independence, activities of daily living and motor function?
	4. Can an individual medical expense and people's medical expenses reduce doing physical therapy?
	5. What is the method of motion learning?
	6. What balance training is of possible practical use for elderly?
	7. Is the physical therapy practiced based on evidence?
	8. Is physical therapy effective for higher brain dysfunction?
	9. What is the effect of the therapeutic exercise in cerebral palsy?

	10. What are the relationships between lifestyle, the quantity of activities of daily living, and physical fitness?<br>11. What research is needed for creating a data base for effective general physical therapy?<br>12. Is the patient satisfied with the provided physical therapy (content or result)?<br>13. Is the form of the clinical practice an ideal way in a physical therapy education?
Rushton & Moore, 2010[3]	**Professional development**<br>1. What are the clinical reasoning processes used in OMT?<br>2. What approaches in education assist in the development of clinical reasoning skills?<br>3. What is the efficacy of teaching OMT techniques?<br>4. What is the patient's experience of OMT?<br>5. What is the effectiveness of training to enhance the validity of palpation skills?<br>6. What are the existing models of postgraduate education in OMT?<br>**Epidemiology**<br>1. What are the descriptive patterns of musculoskeletal disorders?<br>**Normative data collection**<br>1. What are the effects of stretching?<br>2. What is the normal response to a range of examination tests?<br>**Reliability of assessment tools theme**<br>1. What is the intra-rater reliability of a broad range of assessment tools?<br>2. What is the inter-rater reliability of a broad range of assessment tools?<br>3. What is the accuracy of a broad range of assessment tools?<br>**Validity of assessment tools**<br>1. What is the face validity of a broad range of assessment tools?<br>2. What is the content validity of a broad range of assessment tools?<br>3. What is the concurrent validity of a broad range of assessment tools?<br>4. What is the sensitivity of a broad range of assessment tools?<br>5. What is the specificity of a broad range of assessment tools?<br>6. What is the validity of new clinical prediction rules for treatment outcome?<br>7. What is the predictive value of a broad range of assessment tools?<br>**Outcome measures**<br>1. How is patient satisfaction evaluated?<br>2. What factors contribute to patient satisfaction?<br>3. What is the clinical and linguistic validation of existing questionnaires?<br>4. What is the clinical utilisation of various outcome measures?<br>5. What performance based outcome measures are most appropriate for mechanical neck disorders?<br>6. What is an appropriate functional testing outcome measure for use in LBP?<br>**Examination, assessment and diagnosis**<br>1. What are the criteria for diagnosis of different presentations?<br>2. What are the physical findings in patients with vertigo?<br>3. What is the relevance and use of red flags in the management of musculoskeletal disorders?<br>4. What is the relevance and use of yellow flags in the management of musculoskeletal disorders?<br>**Classification/subgroups/profiling of common syndromes theme**<br>1. What are the common physical/subjective/bio-psychosocial characteristics of patient sub groups eg; acute low back pain, chronic low back pain, whiplash associated disorder, tennis elbow, OA hip etc?<br>2. What are the characteristics of the sub group populations responding or not responding to OMT?<br>3. What factors appear to predict outcome of care in individual subgroups?<br>**Mechanism of action of treatment**<br>1. What are the effects of physical activity and exercise on sub populations with musculoskeletal disorders?<br>**Evidence based practice**<br>1. What are the clinical guidelines for the assessment and management of shoulder, knee and ankle problems?<br>2. What is the impact of evidence within OMT?<br>3. What strategies work in helping to integrate evidence into scientific practice?<br>**Patient focused research**<br>1. What are patient expectations of OMT service delivery?<br>2. What are the quality of life issues affecting treatment outcome?<br>3. What factors determine patient satisfaction with OMT?<br>4. What are the pain experiences of patients with acute/chronic low back pain?<br>5. What are the patient's experiences of the treatment of chronic pain?<br>6. What is the influence of patient expectations on OMT treatment and outcomes?<br>7. What are the influences of education on patients with acute/chronic low back pain?
McDonough, 2011[4]	1. An exploration of the factors associated with adherence to exercise and physical fitness programmes<br>2. An examination of the role of exercise in improving mental health of mild/moderate depression<br>3. An investigation into how exercise capabilities should be assessed dependent on disease state including the identification of an exercise prescription

	4. An exploration of the impact of the pressure of targets, waiting lists and the volume of repeat referrals on achieving intervention outcomes that reflect the needs and expectations of patients<br>5. Identification of optimal duration and intensity of treatment and engagement with patients linked to outcomes<br>6. Cost benefit analysis of the provision of services<br>7. More effective incorporation of health economics within future research design<br>8. To research the benefits of physical therapy intervention in promoting an enablement ethos with chronic conditions<br>9. An exploration of the relationship between skill mix and clinical outcomes<br>10. Identification of areas for development in the structure of how therapy is provided – self-management in adults, parent-led therapy in children, and group therapy versus one-to-one approaches<br>11. Research designed to assess the impact of physical activity on health and wellbeing<br>12. The effectiveness of exercise interventions in lymphoedema management<br>13. Assessing the effectiveness of treatments in the management of chronic pain including exercise, acupuncture, education, hypnosis and biopsychosocial approaches<br>14. An assessment of the effectiveness of interventions in the management of back pain including traction, manual therapy and core stability strategies<br>15. Contrasting the clinical effectiveness of the use of classes with one to one treatment approaches<br>16. An investigation into the benefits of exercise based rehabilitation of soft tissue injury<br>17. Evaluation of the role of exercise in cancer rehabilitation. – intensify/frequency etc.<br>18. Research into the use of functional tests in assessment<br>19. An exploration of optimal assessment and treatment times for physical therapy appointments – do longer appointment times result in better long term outcomes?<br>20. The impact of exercise intensity on symptom management and recovery in long term conditions<br>21. The effectiveness of individualised development care for preterm infants born at less than 32 weeks gestation
Goldstein et al., 2011[5]	**Basic science research**<br>1. Identify how genetic, anatomical, biomechanical, physiological, or environmental factors contribute to excessive stress, injury, or abnormal development of body tissues and systems.<br>2. Determine if modifiable genetic, anatomical, biomechanical, physiological, or environmental factors can decrease risk of excessive stress, injury, or abnormal development of body tissues and systems.<br>3. Examine the effects of physical therapy interventions that are provided independently or in combination on cellular structural properties and physiological responses of healthy, injured, or diseased body tissues.<br>4. Investigate the factors that modify the response to physical therapy intervention and positive tissue adaptation (eg, genetic, functional, structural, psychosocial, and physiological factors).<br>5. Determine the optimal dose of physical therapy interventions (frequency, duration, intensity) to achieve optimal cellular and physiological adaptation/response of body tissues and systems.<br>6. Examine skill acquisition and motor development in individuals with movement disorders.<br>7. Examine the relationship between biomarkers and impairments in body structure and function, limitations in activity, and restrictions in participation. (Biomarkers are any tools used to identify and quantify biologic responses).<br>8. Define the role for physical therapy in the maturation and 5odelling of genetically engineered tissues.<br>9. Determine the mechanisms by which physical therapy interventions modify disease and age-related or injury-induced changes in normal cellular structure and function using appropriate human and animal models.<br>10. Develop new physical therapy interventions to promote tissue growth and adaptation.<br>**Clinical Research**<br>1. Determine the relationships among levels of functioning and disability, health conditions, and contextual factors for conditions commonly managed by physical therapists (eg, International Classification of Functioning, Disability and Health).<br>2. Develop and evaluate models of health and disability to guide the investigation, prevention, and treatment of health conditions relevant to physical therapy.<br>3. Identify factors that predict the risks of, or protection from, health conditions (injury, disorders, and disease).<br>4. Examine the impact of health promotion interventions that include the involvement of physical therapists on activity and participation of individuals with movement disorders.<br>5. Evaluate or develop effective interventions to prevent or reduce the risk of disability associated with common health conditions.<br>6. Determine the effects of interventions provided by physical therapists to address secondary prevention in patients/clients with chronic diseases (eg, diabetes, obesity, arthritis, neurological, other disorders).<br>7. Determine the physical therapist's role and impact in contemporary delivery models on prevention of diseases and their secondary side effects.<br>8. Identify technologies to assist physical therapists in developing prevention approaches that optimize outcome.<br>9. Develop and evaluate effective patient/client classification methods to optimize clinical decision making for physical therapist management of patients/clients.<br>10. Identify criteria for progression in levels of care, activity, or participation of the patient/client.<br>11. Identify thresholds for adequate physical function to optimize outcomes and prevent injury.<br>12. Identify contextual factors (eg, personal and environmental) that affect prognosis.<br>13. Identify technologies to assist physical therapists in determining patient/client classification.

14. Determine predictors of recovery from adverse effects associated with medical or surgical treatment.
15. Determine the effectiveness and efficacy of interventions provided by physical therapists across relevant domains of health.
16. Determine interactions among interventions provided by physical therapists.
17. Determine the effectiveness and efficacy of interventions provided by physical therapists delivered in combination with other interventions (eg, medical, surgical, or biobehavioral interventions).
18. Determine the effects of frequency, duration, intensity, and timing of interventions provided by the physical therapist.
19. Develop and test the effectiveness of physical therapist interventions for primary and secondary conditions or disability.
20. Develop and test the effectiveness of physical therapist interventions to optimize treatment outcomes for specific subgroups of patients/clients.
21. Develop and test the effectiveness of decision support tools to facilitate evidence-based physical therapist decision making.
22. Develop and test the effectiveness of methods to improve patient/client adherence to the plan of care and self-management.

Education/Professional Development

1. Evaluate the effect of physical therapist post professional specialty training on clinical decision making and patient/client outcomes.
2. Determine the best methods to foster career development and leadership in physical therapy.
3. Determine the optimal criteria for board certification.
4. Evaluate the effect of clinical education models on clinical outcomes, passing rates on the National Physical Therapy Examination, and employment settings after graduation.
5. Determine the impact of professional-level physical therapist education on professional behaviors.
6. Assess the effectiveness of models of professional education on clinical performance.
7. Determine the relationship between student cultural competency and clinical decision making.
8. Evaluate the effectiveness of different methods used to improve cultural competence.
9. Develop and evaluate the most effective methods for facilitating physical therapist acquisition and use of available information resources for evidence-based practice.
10. Evaluate the skills needed by practitioners to provide optimal patient/client care, patient/client advocacy, and cost-effective care.

Epidemiology

1. Examine the incidence, prevalence, and natural course of health conditions (disorders, diseases, and injuries) commonly managed by physical therapists.
2. Examine the incidence, prevalence, and natural course of impairments of body functions and structure, activity limitations, and participation restrictions associated with health conditions commonly managed by physical therapists.
3. Investigate the effects of contextual factors (eg, personal and environmental) on the effectiveness of interventions provided by physical therapists.

Health Services Research/Policy

1. Perform economic evaluation of specific physical therapy interventions.
2. Evaluate the effect of physical therapy service delivery models on economic and patient/client outcomes and consumer choice.
3. Determine the relationship between documentation and payment.
4. Evaluate the comparative cost and/or cost-effectiveness of specific physical therapy interventions compared with or in combination with other interventions.
5. Investigate factors that influence patient/client choices when selecting a health care provider or making treatment decisions.
6. Develop and evaluate new methods for incorporating patient/client values and expectations into the decision-making process.
7. Evaluate the effectiveness of shared clinical decision-making schemes between the patient/client and therapist on clinical outcomes and costs.
8. Establish the extent to which physical therapists deliver services in accordance with recommended guidelines for specific conditions and its impact on outcomes.
9. Determine disparities in the access to and provision of physical therapy and their impact on outcomes.
10. Examine the interaction among access, culture, and health literacy on physical therapy outcomes.
11. Examine the cultural competence of physical therapists and physical therapist assistants and its impact on intervention.
12. Develop innovative medical informatics applications for physical therapy and assess their impact on clinical decision making.
13. Investigate the influence of health policies on practice patterns and outcomes.
14. Evaluate methods to enhance adherence to recommended practice guidelines.
15. Assess the impact of continuity of physical therapy services on outcomes.
16. Describe patterns of physical therapy use and identify factors that contribute to variation in utilization.

Workforce

1. Examine the effects of staffing patterns on the outcomes of physical therapy.

	2. Assess productivity of physical therapists in various settings and identify factors (eg, use of extenders, mandates) that contribute to variations in productivity.<br>3. Identify and test the best methods to assess past, current, and future demand and unmet needs for physical therapy.<br>4. Identify the demand for services among populations underserved by physical therapists.<br>5. Determine factors that contribute to the attractiveness of practicing in various settings and geographic regions.<br>6. Determine factors that contribute to the retention of physical therapists across various settings and geographic regions.<br>7. Determine the effectiveness of recruitment and retention initiatives in reducing the gap between supply and demand in various practice settings.<br>8. Identify variables that influence the decision of whether or not to enter the physical therapy profession.<br>9. Assess the impact of expanded scope of practice on supply and demand.<br>10. Investigate the relationship between the distribution of physical therapists and population health outcomes.<br>11. Examine the effects of workforce issues on career pathways (eg, participation in residency, fellowship, research training).<br>12. Examine the effects of participation in extended clinical training experiences on workforce.<br>**Measurement Development and Validation**<br>1. Develop or adapt measures of effectiveness and impact of physical therapy at the community level.<br>2. Develop new tools or refine existing tools to measure the impact of physical therapy on activity, participation, and quality of life.<br>3. Provide evidence to guide selection and interpretation of measurement tools for specific purposes, conditions, and populations.<br>4. Develop and test a minimum set of measures to evaluate the process and clinical outcomes for specific conditions and populations.<br>5. Develop reliable and valid measures of cultural competence of physical therapy providers and students.<br>6. Determine how contemporary technology (eg, ultrasound, gene array, magnetic resonance) can be used to measure the effects of injury/disease and physical therapy intervention on body structure and function.<br>7. Determine optimal measurement methods to enhance clinical decision making for specific conditions and populations
Rankin et. al, 2012[6]	**Musculoskeletal**<br>1. Exploration of interventions/strategies to increase patients' adherence to/concordance with exercise programmes (adherence to exercise programmes)<br>2. Exercise as medicine: prescription of exercise (i.e. type, dosage, environment) for patients with chronic, long-term musculoskeletal conditions (exercise prescription)<br>3. The clinical and cost effectiveness of physical therapy for patients with patellofemoral pain (effectiveness of physical therapy management: lower body quadrant problems)<br>4. Development and evaluation of physical activity programmes for older people (effectiveness of physical therapy management: specific conditions)<br>5. The role of physical therapists in promoting exercise and physical activity – preventative healthcare, not only treatment (exercise prescription)<br>6. Effectiveness of graduated rehabilitation for patients with shoulder pain (effectiveness of physical therapy management: upper body quadrant problems)<br>7. What are the benefits of physical therapy rehabilitation for patients with low back pain? (effectiveness of physical therapy management: low back pain)<br>8. What is the impact and value of physical therapy on return to work for people with musculoskeletal conditions? (return to work)<br>9. How do we improve the outcomes for people with shoulder impingement syndrome (effectiveness of physical therapy management: upper body quadrant problems)<br>10. Management of anterior knee pain in children and in adolescents (effectiveness of physical therapy management: paediatric conditions)<br>11. Motivating the demotivated to exercise (adherence to exercise programmes)<br>12. What is the optimum exercise intervention for individuals with osteoarthritis of the knee? (effectiveness of physical therapy management: osteoarthritis)<br>13. Harnessing the potential of routine use of Patient Reported Outcome Measures in physical therapy practice (outcomes of care)<br>14. Effectiveness of self-referral to physical therapy for musculoskeletal conditions (service provision: self referral to physical therapy)<br>15. Do workplace modifications and physical therapy treatments facilitate early return to work, with cost benefits to the employer? (return to work)<br>16. Early physical therapy for acute low back pain in a working population (effectiveness of physical therapy management: low back pain)<br>17. Developing the right tools to assess outcomes of physical therapy interventions (outcomes of care)<br>18. Effectiveness of early physical therapy intervention on anterior knee pain in children and young people (effectiveness of physical therapy management: paediatric conditions)<br>19. The clinical and cost effectiveness of physical therapy for anterior cruciate ligament injuries (effectiveness of physical therapy management: lower body quadrant problems)

20. The role of physical therapy in preventing the next episode of spinal pain; and to what extent does adherence influence the outcome of physical therapy management regimes (public health)
21. Comparing physical therapist-led vs exercise professional-led exercise prescription (exercise prescription)
22. New ways of delivering falls prevention therapy for older people (service provision)
23. Developing and testing a brief screening tool that can assist physical therapists to explore obstacles to return to work (return to work)
24. Effectiveness of rehabilitation compared to surgery for anterior cruciate ligament rupture (effectiveness of physical therapy management: lower body quadrant problems)

Neurology

1. What is best practice in the rehabilitation of the upper limb in patients with stroke with respect to timing, content and dosage? (effectiveness of physical therapy management: post stroke)
2. Dose response studies – how do intensity, frequency and duration of physical therapy interventions relate to outcomes for different stages of stroke recovery and for other neurological conditions? (parameters of intervention)
3. What is the role, content and effectiveness of self-management strategies in long term neurological conditions? (effectiveness of specific interventions: self-management)
4. What are the benefits of stroke rehabilitation on function and quality of life in the longer term (>6 months and >1 year post stroke)? (effectiveness of physical therapy management: post stroke)
5. Is self-practice of repetitive, high intensity, task-specific activities beneficial to the motor recovery of patients with stroke? (effectiveness of physical therapy management: post stroke)
6. Specific therapy interventions in the community setting for the continuing support of people with long term neurological conditions (effectiveness of physical therapy management: other neurological conditions)
7. What are the benefits of aerobic exercise for people with long term neurological conditions, and what are effective service delivery models? (exercise/physical activity)
8. Effectiveness of exercise and fitness programmes for stroke survivors, including the long term benefits on function, quality of life and subsequent stroke prevention (exercise/physical activity)
9. The efficacy of therapy interventions for patients early after stroke onset (effectiveness of physical therapy management: post stroke)
10. Cost effectiveness of service delivery models to increase the intensity of therapy in stroke units (service provision: post stroke)
11. The role of assistive technologies in the restoration of motor function in people with neurological conditions (effectiveness of specific interventions: adjuncts/equipment/assistive technologies)
12. Demonstrating the value of physical therapy interventions in maintaining mobility for patients with Multiple Sclerosis (MS) (effectiveness of physical therapy management: multiple sclerosis)
13. What role should physical therapy play in the management of people with long-term neurological conditions? (effectiveness of specific interventions)
14. Stroke rehabilitation for residents of care homes (effectiveness of physical therapy management: post stroke)
15. The development of appropriate service delivery models for use in long term (service provision: post stroke)
16. Investigation of the efficacy of physical therapy for patients with different types of ataxia (effectiveness of physical therapy management: ataxia)
17. Promotion of physical fitness and activity for children and young adults with Cerebral Palsy and Neurodisability (effectiveness of physical therapy management: paediatric neurological conditions)
18. Implementing intensive repetitive practice for patients with stroke (service provision: post stroke)
19. What is the optimal frequency and timing of physical therapy interventions for children with cerebral palsy? (effectiveness of physical therapy management: paediatric neurological conditions)
20. Contracture management: how should physical therapists treat and advise patients with neurological conditions to prevent or reduce contracture development that impedes functional recovery? (effectiveness of specific interventions: postural)
21. What is the impact and cost-effectiveness of post-stroke rehabilitation for the long term stroke survivor? (parameters of intervention)
22. What are the components and necessary timing of effective complex postural management for patients with severe neurological impairment, such as late stage multiple sclerosis or severe traumatic brain injury? (effectiveness of specific interventions: postural)
23. Establishing an effective at-home exercise programme, focusing on improving balance, for patients with progressive ataxia (effectiveness of physical therapy management: ataxia)
24. What role do physical interventions play in combination with botulinum toxin (BTX) in the management of spasticity in patients following central neurological damage? (effectiveness of specific interventions)
25. Access to physical activity and fitness activities for people with long term neurological conditions (exercise/physical activity)
26. The effectiveness of modern therapy adjuncts e.g. functional electrical stimulation, Seabo Flex, constraint induced therapy (effectiveness of specific interventions: adjuncts/equipment/assistive technologies)
27. The recovery profile of patients with spinal cord injury (SCI) and the influence of early mobilisation (effectiveness of physical therapy management: spinal cord injury)

28. Efficacy of cardiovascular fitness and energy expenditure markers in the initial rehabilitation and in the long term management of patients with spinal cord injury (SCI) (effectiveness of physical therapy management: spinal cord injury)
29. Improving adherence of patients with Multiple Sclerosis (MS) in remaining physically active following a physical therapy intervention (effectiveness of physical therapy management: multiple sclerosis)
30. Effectiveness of self-management programmes (SMPs) for patients with stroke (effectiveness of specific interventions: self management)
31. Can physical therapy reduce falls in patients with Parkinsons Disease? (effectiveness of physical therapy management: other neurological conditions)
32. Exploring the best strategies for motor relearning in patients with cognitive or language deficits (effectiveness of physical therapy management: other neurological conditions)
33. Self-management in cerebral palsy: at the time of transition to adult services, would education in self management of physical problems associated with cerebral palsy improve the quality of life and socio-economic independence of young adults with cerebral palsy? (transition from paediatric to adult services)
34. The optimum level of physical therapy for children and young people with neurological conditions (parameters of intervention)
35. The effect of intermittent rehabilitation over a long-term period on patients' function and participation in society (parameters of intervention)
36. How can physical therapists help to improve adherence to physical activity programmes developed for patients with neurological conditions in the community? (exercise/physical activity)
37. Potential prognostic indicators for therapeutic interventions for patients with stroke (tailored treatment)
38. The effects and experiences of physical activity interventions for non-ambulatory patients with stroke or other long-term neurological conditions (exercise/physical activity)
39. Effectiveness of long-term stroke support services for stroke survivors and their carers (service provision: post stroke)
40. An evaluation of exercises and exercise devices for patients with progressive ataxia using wheelchairs (effectiveness of physical therapy management: ataxia)
41. Effectiveness of gait rehabilitation for patients with stroke (effectiveness of physical therapy management: post stroke)
42. Demonstrating the value of physical therapy for patients with Multiple Sclerosis (MS) following a relapse (effectiveness of physical therapy management: multiple sclerosis)
43. Falls management in people with long term neurological conditions, especially with stroke and Multiple Sclerosis (guidelines/policy: development, implementation and impact)

Cardiorespiratory

1. Does access to an emergency on-call physical therapy service improve patient outcomes? (service provision)
2. What are the benefits, cost-effectiveness and long term impact on patient outcomes of delivering 7-day physical therapy services within secondary care? (service provision: 7-day working)
3. The effect of a physical therapist-led early mobility programme in Intensive Therapy Units (ITUs) on patients' long-term outcomes of function, mobility and quality of life (effectiveness of physical therapy management: critically ill patients)
4. Long term benefits and cost effectiveness of different models of physical therapy intervention for new lower limb amputees (effectiveness of physical therapy management: amputees)
5. Comparative work on the role of the physical therapist in post-critical care rehabilitation and follow-up clinics, to look at long-term outcomes and possible predictors of functional outcome (effectiveness of physical therapy management: critically ill patients)
6. Investigating interventions which could enhance recovery in patients with critical illness (effectiveness of physical therapy management: critically ill patients)
7. Pulmonary rehabilitation intervention in patients with early stage Chronic Obstructive Pulmonary Disease (COPD) (effectiveness of cardiopulmonary rehabilitation)
8. The efficacy of the cough assist technique in patients with neuromuscular disease (effectiveness of specific interventions: airway clearance)
9. Exercise interventions for patients with critical illness: feasibility and physiological and functional outcomes (effectiveness of specific interventions: exercise/physical activity)
10. Rehabilitation programmes in palliative care (effectiveness of specific interventions)
11. Short and long term effectiveness of pulmonary rehabilitation (PR) initiated either during or post admission for an exacerbation of Chronic Obstructive Pulmonary Disease (COPD) (effectiveness of cardiopulmonary rehabilitation)
12. The effects of early mobilisation and rehabilitation for paediatric patients in the Intensive Care Unit (ICU) (effectiveness of physical therapy management: critically ill patients)
13. What are the main modalities of treatment for patients with phantom pain after amputation in the UK? (effectiveness of physical therapy management: pain after amputation)
14. Manual chest physical therapy techniques for secretion clearance for patients who are intubated and mechanically ventilated (effectiveness of physical therapy management: critically ill patients)
15. What are the service needs of patients with end stage respiratory disease? (patient centred practice)
16. The frequency, intensity and timing of exercise required to optimise rehabilitation for patients with critical illness (effectiveness of specific interventions: exercise/physical activity)
17. Improving adherence to chronic disease management services such as pulmonary rehabilitation and exercise programmes for patients with chronic lung disease (effectiveness of specific interventions: exercise/physical activity)

18. What is the efficacy of self management and educational interventions for patients with respiratory compromise? (adherence)
19. Effectiveness of physical therapist-led exercise classes for people with long term conditions (tailored treatment)
20. Does the use of risk prediction models to determine which patients receive post-operative physical therapy (effectiveness of specific interventions: exercise/physical activity)
21. Benefits of physical activity and exercise in adult patients with congenital heart disease (the role of the physical therapist)
22. The role of physical therapy in the care of patients with dementia to assist in the maintenance of functional independence (effectiveness of specific interventions: airway clearance)
23. Investigating the physiological mechanisms of airway clearance interventions, evaluating different techniques, and short and long term outcomes in patients with non-cystic-fibrosis bronchiectasis (effectiveness of specific interventions: exercise/physical activity)
24. Comparing the effectiveness of different models of pulmonary rehabilitation (effectiveness of cardiopulmonary rehabilitation)
25. Cost effectiveness and long term benefits of physical therapy management of patients with acute exacerbations of Chronic Obstructive Pulmonary Disease (COPD) (service provision)
26. Long term effectiveness of physical therapy for patients with cystic fibrosis (effectiveness of physical therapy management: cystic fibrosis)
27. Efficacy of exercise training for low functioning and high risk patients (effectiveness of specific interventions: exercise/physical activity)
28. Developing a better understanding of the reality of how amputees wear and use their limb in the normal day (effectiveness of physical therapy management: amputees)
29. Developing outcome measures to evaluate the effectiveness of physical therapy for patients in palliative care (outcome measures)
30. The effectiveness of physical therapy service provision for young men with Duchenne's muscular dystrophy at the point of transition from paediatric to adult services (service provision)

Wellbeing

1. Effectiveness of programmes to change physical activity behaviour for people with long term conditions (physical activity)
2. Developing effective collaborations with third sector exercise/activity providers for people with chronic musculoskeletal pain (physical therapy role in exercise intervention)
3. What training and development is needed by physical therapists at pre- and post-registration to ensure they can be effective in facilitating health behaviour change? (education/continuing professional development)
4. Optimum levels of exercise in the treatment and prevention of mental health problems, for example, depression and dementia (effectiveness of exercise: managing depression)
5. Evidence to support the physical therapist's role in delivering exercise programmes in a variety of settings (physical therapy role in exercise intervention)
6. Physical therapy management of chronic musculoskeletal dysfunction in the older patient (effectiveness of physical therapy)
7. Promoting engagement in physical activity for people with long-term conditions (physical activity)
8. Physical therapy interventions for people with dementia in acute hospital settings (effectiveness of physical therapy management of dementia)
9. Effectiveness of physical exercise in the recovery of patients with chronic obstructive pulmonary disease (COPD) after acute exacerbations (effectiveness of exercise intervention)
10. How can physical therapists educate and influence patients in the need to engage more regularly in physical activity? (physical activity)
11. Exploring barriers to behaviour change in the management of long term conditions (health behaviour change)
12. Physical therapy and cognitive behavioural therapy (CBT) (Effectiveness of physical therapy)
13. Do physical therapists have the skills to advise patients on exercising for health? (physical therapy role in exercise intervention)
14. Effectiveness of early supported discharge in getting patients out of hospital (service provision)
15. Exploring access to appropriate physical therapy and rehabilitation for patients with dementia disorders in the UK, to maintain/improve mental and physical health and wellbeing and independence (effectiveness of physical therapy management of dementia)
16. How do we integrate models of working with fitness instructors, physical therapists and GPs to get the best outcomes for patients? (physical therapy role in exercise intervention)
17. Optimising the self-management support given by physical therapists to patients with chronic diseases (health behaviour change)
18. What role does physical therapy play in keeping people with specific conditions out of hospital, for example, those with respiratory conditions, mobility problems or at risk of falling? (physical therapy in public health)
19. Physical therapy management of patients with specific conditions who also have mental health problems (education/continuing professional development)

	20. Effective tools for assessing and treating pain in patients with dementia (effectiveness of physical therapy management of dementia)<br>21. The role of non-specific exercise in management of long term conditions (effectiveness of exercise intervention)<br>22. The role of physical therapy in promoting return to work for people with musculoskeletal and mental health conditions (role of the physical therapist in public health/health promotion)<br>23. Effectiveness of exercise and relaxation interventions for patients with common mental health conditions. (effectiveness of exercise: managing depression)<br>24. Long term effectiveness of providing community rehabilitation services for patients with long term conditions or those recovering from serious illness. (community based physical therapy services)<br>25. Does exercise decrease recurrence and improve survival in patients with cancer? (effectiveness of exercise: patients with cancer)<br>26. The role of physical therapy in 'fit note' schemes. (role of the physical therapist in public health/health promotion)<br>27. Optimum staffing ratios and reasonable waiting times in relation to effective service delivery (service provision)<br>28. What are valid outcome measures in evaluating physical therapy practice and patient benefit? (outcomes of care)<br>29. Optimising the frequency and intensity of exercise for older patients. (effectiveness of exercise: older people)<br>30. How effective are physical therapists in influencing patients to be more active? (physical activity)
Pollock et al., 2012[7]	1. What is the best physical therapy regime for recovery after stroke?
Gierisch et al., 2014[8]	2. What are the comparative safety and effectiveness of usual care nonsurgical therapies (pharmacotherapy, injections, physical therapy/exercise, and weight loss) or combinations of usual care nonsurgical therapies to prevent progression of and disability from OA? Are these effects maintained (i.e., long-term outcomes) over time?
Boney et al., 2015[9]	1. How can preoperative exercise or fitness training, including physical therapy, improve outcomes after surgery?
Morris et al., 2015[10]	1. Does the timing and intensity of 'early' intervention (eg, providing information, physical therapy, speech and language therapy, occupational therapy, etc) alter effectiveness of therapies for infants and young children with neurodisability, including those without specific diagnosis? What is the appropriate age of onset/strategies/dosage/direction of therapy interventions?<br>2. Are any types of physical therapy (eg, Bobath, Neuro-Developmental Therapy, hydro, constraint, strength-training, etc) more or less effective to promote motor functioning in children and young people with neurodisability (eg, cerebral palsy, acquired brain injury)?
Nast et al., 2016[11]	1. Research should develop and evaluate efficient physical therapy treatment methods<br>2. Research should evaluate the influence of treatment type, frequency, intensity and length on socioeconomic and patient relevant outcomes (e.g. improvement of autonomy in older age, reduction of work absence and improvement of quality of life)<br>3. Research should develop physical therapy assessment and diagnosis further, as specification of patients' problems is a prerequisite for tailored, cost-effective treatment<br>4. Research should focus on physical therapy assessment and diagnosis in the area of chronic, highly prevalent diseases (e.g. obesity and chronic back pain)<br>5. Research should develop and evaluate programmes in the field of secondary prevention (e.g. activity promotion and prevention of falls in elderly people and prevention of chronic manifestations of disease)<br>6. Research should develop and evaluate programmes in the field of primary prevention (e.g. movement related health promotion in schools and prevention of back pain)<br>7. Research should address barriers and facilitators within physical therapist–patient interaction<br>8. Research should explore barriers and facilitators within physical therapist–patient interaction<br>9. Research should engage in experiences and behaviours of patients (e.g. motivation, compliance, coping and psychosocial problems)<br>10. Research should further develop and evaluate educational systems tailored best on physical therapy professional education<br>11. Physical therapy should invest in educational research and in the further development and evaluation of curricula<br>12. Research should explore changing requirements of the physical therapy profession (e.g. due to a higher share of the population of older people or increasing amount of chronic diseases)<br>13. Research should explore effects of the shift of the professional education to the level of University of Applied Sciences<br>14. Research should focus on necessary competencies of physical therapists required for the implementation of direct access<br>15. Research should develop and evaluate pilot projects for direct access<br>16. Research should improve knowledge of new technologies and their possible impact on physical therapy (e.g. new surgical techniques, assistive devices and orthoses)<br>17. Research should contribute to the implementation of new technologies for physical therapy (e.g electronic patient file and virtual reality in technology for diagnosis and treatment of movement disorders)<br>18. Research should contribute to the further development of physical therapy continuing education<br>19. Research should focus on evaluating the current system in terms of the future quality in physical therapy practice<br>20. Research should develop multidisciplinary health care networks addressing changing societal needs<br>21. Research should evaluate the effects of physical therapy networks

Rangan et al., 2016[12]	1. Does early mobilisation and physical therapy after shoulder surgery improve patient outcome compared to standard immobilisation and physical therapy?<br>2. Are patients (including older age groups) with rotator cuff tendon tears in their shoulder best treated with surgery or physical therapy?
KNGF, 2017[13]	**Choosing wisely**<br>1. What is the effectiveness of (early) physical therapy on (non-)medical cost-savings and/or substitution of more expensive forms of health care (like surgery or expensive medication)?<br>2. What is the effectiveness of physical therapy on physical functioning, compared to usual care or no intervention (wait-and-see-policy)?<br>3. Which physical therapy interventions that have been proven to be effective need to be (better) implemented, and/or which physical therapy interventions that have been proven to be ineffective need to be (better) de-implemented in daily practice?<br>4. Which core outcome sets of patient-relevant and crucial (generic if possible) outcome measures and minimally clinically relevant improvements should be used by physical therapists in daily practice?<br>5. Which criteria (generic if possible) to start or to end a treatment of physical therapy should be used by physical therapists in daily practice?<br>6. What is the effectiveness of physical therapy on work absence, return to work and work-related (societal) costs?<br>**Tailor-made treatment**<br>1. Which adaptations in physical therapy interventions are necessary to be effective in complex patient groups, like patients with multi-morbidity, intellectual disability or frailty?<br>2. What is the optimal content, intensity and duration of physical therapy interventions and for which specific patients ('personalized care') or specific subgroups ('stratified care') to optimize the size of effects?<br>3. What is the added value of behavioral interventions within the physical therapy treatment on patient adherence and sustained treatment effects, and what are the required competences of physical therapists to provide this?<br>**Technology in health care**<br>1. What is the feasibility and added value of technological devices for physical therapists, aiming at optimizing the diagnostic or therapeutic process?<br>2. What is the feasibility and added value of 'internet-based care' or 'blended care', aiming at enhancing patient adherence and sustained treatment effects, compared to completely supervised physical therapy, usual care or no intervention ('wait-and-see-policy')?<br>3. What is the possible role of 'big data', collected through technological devices, in monitoring health (reductions) and physical functioning in specific patient groups, or in identifying diseases in an early phase in health people?
CSP, 2018[14] and Rankin et al., 2020[15]	Top 10 (Rankin et. al)<br>1. When health problems are developing, at what point is physical therapy most/least effective for improving patient results compared to no physical therapy? What factors affect this?<br>2. When used by physical therapists, what methods are effective in helping patients to make health changes, engage with treatment, check their progress, or manage their health after discharge?<br>3. What are the best ways to deliver physical therapy services to meet patients' needs and improve outcomes for patients and services?<br>4. To stop health problems occurring or worsening, what physical therapy treatments, advice or approaches are safe and effective? Where more than one treatment/approach works, which work best and in what dose?<br>5. What are patients' expectations regarding recovery, how do these compare to physical therapists' views and, where recovery is not possible, how is this managed?<br>6. How does waiting for physical therapy affect patient and service outcomes?<br>7. What parts of physical therapy treatments cause behaviour change or physical improvement?<br>8. What approaches are effective for enabling parents, relations or carers to support physical therapy treatment or to help patients to manage their own health problem?<br>9. How is patient progress and/or the results of physical therapy treatment measured? How is service performance measured and checked?<br>10. How can access to physical therapy be improved for groups who have reduced access?<br>Remaining questions (CSP 2018)<br>1. How does the amount of physical therapy received affect results for patients and services? What are optimal session lengths, frequency and duration of treatment?<br>2. What types of exercises, doses and methods of delivery are effective in stopping health problems occurring or worsening?<br>3. What do the people who fund services and internal budget holders understand about the role of physical therapy and how do they make funding decisions?<br>4. How well do patients recall physical therapy advice and to what extent do patients follow this advice?<br>5. What's the availability of physical therapy services nationally, how does this compare between specialisms, countries, or to documented need? What affects service availability across the UK?<br>6. When trying to improve patient and service outcomes, what types of exercises, doses and methods of delivery are effective?<br>7. What are the physiological effects of different physical therapy treatments?

	8. What methods do physical therapists use to treat patients, to help them gain skills to manage their condition and to use them in their daily lives?<br>9. Do staffing levels and skill mix impact patient and service outcomes? What are the best staffing levels and skill mixes in different areas of physical therapy and how do these compare to current staffing provision?<br>10. How do physical therapists decide on what their treatment plans include and/or when to refer on? What influences the types of evidence they use?<br>11. What factors predict the onset of health problems, patient responses to physical therapy or their abilities to make health changes/self-manage? Which patients (if any) are likely to benefit most/least from physical therapy?<br>12. What are patients offered nationally in terms of treatment sessions, appointment times and follow-on care? How is it checked that this is enough?<br>13. What training is available to physical therapists for developing their skills either working with different conditions or using more specialist approaches?<br>14. How are different physical therapy services provided, staffed and accessed across the UK and what influences this?<br>15. What do patients expect of physical therapy and understand in terms of remaining healthy, their condition and their role in self-management?
Gomes et al., 2018[16]	1. Utilisation of evidence-based practice (barriers, adherence, results from implementation strategies)<br>2. Cost effectiveness of various physical therapy modalities, physical therapy vs other interventions and different response models<br>3. Mechanisms that justify the effects of physical therapy interventions (manual therapy, exercise)<br>4. Effectiveness of physical therapy in musculoskeletal symptoms and conditions<br>5. Physiological effects (nervous system; physiological markers) of physical therapy interventions (manual therapy, education, exercise)<br>6. Effectiveness of physical therapy in the prevention of pain/injury/musculoskeletal conditions<br>7. Effectiveness of interventions/strategies to promote adherence to the intervention and self-management of the musculoskeletal condition<br>8. Definition of parameters/dose to optimize effects of interventions<br>9. Identification and assessment of risk factors for musculoskeletal injuries/conditions<br>10. Patient satisfaction with recommended physical therapy/interventions
Fernandez et al., 2018[17]	1. What is the best physical therapy and/or occupational therapy regime for adults during their in-hospital recovery from a fragility fracture of the lower limb?<br>2. What is the best physical therapy and/or occupational therapy regime for adults during out-of-hospital recovery from a fragility fracture of the lower limb?<br>3. What are the best physical therapies to treat adults with a fear of falling after a lower limb fragility fracture?
Wilson et al., 2019[18]	1. Understanding the impact of gait dysfunction on participation in daily life<br>2. Development and validation of gait-specific outcome measures<br>3. Understanding the impact of gait dysfunction on community integration<br>4. Development and validation of acquired brain injury-specific gait treatments<br>5. Understanding impairments that contribute to gait dysfunction<br>6. Development and validation of technology for treatment of gait impairments
Moerchen et al., 2020[19]	1. Identify the relevant pediatric physical therapy content (knowledge, skills, abilities, experiences, behaviors, attitudes, etc) that should be included in pediatric physical therapy education<br>2. Identify effective methods of measuring outcomes of educational experiences related to pediatric physical therapy that demonstrate that students or practitioners have acquired the identified relevant knowledge, skills, abilities, experiences, behaviors, attitudes, etc.<br>3. Identify effective teaching and learning methods/strategies for the instruction, practice, and evaluation of skills, behaviors, and attitudes that are meaningful to pediatric physical therapy outcomes (including but not limited to experiential learning, clinical reasoning, simulation, computer assisted)<br>4. Identify indicators of readiness for pediatric physical therapy practice<br>5. Identify the preferred amount of experiential learning that is meaningful to pediatric physical therapy education outcomes<br>6. Identify the effect (on curriculum, outcomes, student learning/readiness for practice, etc) of the published Essential Competencies in Entry-Level Pediatric Physical Therapy Education<br>7. Identify definitions and indicators for excellence in pediatric physical therapy education<br>8. Identify the expectations of employer/employee readiness for pediatric physical therapy practice and what effect these expectations have on curricula<br>9. Identify the benefits and challenges of differences in curricular content (depth and breadth) on pediatric physical therapy education outcomes<br>10. Determine the effectiveness of strategies/methods/practices for promoting and enhancing knowledge translation, continued competency, and advanced practice in pediatric physical therapy<br>11. Identify the benefits and challenges of different curricular models on pediatric physical therapy education outcomes<br>12. Determine the effectiveness of current strategies/methods/practices for preparing individuals as educators, including both academic and clinical, for pediatric physical therapy education

APTA, 2021[20]	**Population health research**<br>1. Disparities: Investigate health equity in rehabilitation (disparities across race and ethnicities, various age groups, cultures, and socioeconomic status) — and its impact on access to care and outcomes, reducing clinician biases, and institutional and community level oppression<br>2. Social determinants of health: Investigate the mechanisms and mediators by which upstream social determinants of health (food insecurity, physical environment, access-to-care, education, employment/working conditions, transportation barriers, early child development, etc.) shape the development of disparities in impairments, activity limitations, participation restrictions, and poor rehabilitation outcomes, in order to identify targets for multilevel intervention<br>3. Interventions: Investigate the impact of physical therapy on population outcomes (health and wellness) across the lifespan; including total cost of care, quality of life, reductions in disability, and reduction in the burden of care.<br>**Clinical research**<br>1. Telehealth: Determine the effectiveness of telehealth delivery, clinical examination via telehealth, and patient engagement strategies, and identify patient populations most likely to benefit from telehealth<br>2. Value: Determine value (cost-effectiveness, impact on longer-term clinical outcomes, and quality of life) of physical therapy compared with nonrehabilitation treatments for clinical conditions appropriate for physical therapy<br>3. Treatment: Determine the effects of physical therapist interventions in addressing secondary prevention and health promotion in individuals with chronic diseases (diabetes, obesity, arthritis, neurological, and other disorders)<br>**Health services research**<br>1. Delivery models: Explore cost analysis and assess outcomes of delivery of care models (one-on-one versus multiple patients per provider versus team based) and facility ownership (outpatient corporate versus outpatient independently owned versus outpatient hospital-affiliated versus inpatient hospital based) and payment models (insurance versus cash pay)<br>2. Utilization and cost: Explore the financial benefits for physical therapy. Compare utilization and costs (outcomes/costs) of the numerous practice areas for physical therapists (identifying where physical therapists add value to the system, where physical therapists add additional cost, where physical therapy services add redundancy, etc.)<br>3. Payment and insurance: Explore the impact of various payment models and insurance providers on patient and clinical outcomes and downstream costs and utilization
APTA Pediatrics 2021[21] and Bhat et al., 2022[22]	**Basic Science Research**<br>1. Explore factors that affect growth and development of muscles, bones, neural networks, and other tissues and systems that contribute to movement<br>2. Explore the mechanisms of tissue damage and repair in the musculoskeletal, neuromuscular and cardiorespiratory systems<br>3. Describe development in infants and children that are typically developing, at risk for movement-related disorders, or are diagnosed with movement-related disorders<br>4. Investigate critical/sensitive periods for neuroplasticity and motor development (e.g. in infancy or after neural injury)<br>5. Examine relationships between motor development and other domains of child development (e.g. cognitive, social, emotional, and language)<br>6. Examine brain-behavior relationships during functional behaviors<br>7. Explore physical activity/participation levels in children and adults<br>**Clinical research**<br>1. Identify impairments in children with or at risk for movement-related disorders - Explore factors associated with movement-related impairments<br>2. Determine the effects of PT interventions and other factors such as nutrition on:<br>a. skeletal muscle and tendon (e.g. development and modification of muscle architecture, strength, power, muscle and tendon length, activation patterns, and recovery from injury or surgery)<br>b. bones and joints (e.g. development and modification of bone density and architecture, infant head shape, alignment of joints, and recovery from injury or surgery)<br>c. central and peripheral nervous system (e.g. development and modification of neural pathways and networks, activity-dependent neural adaptation, regeneration, restoration and compensatory changes after nervous system damage)<br>d. the cardiorespiratory system, metabolism and caloric balance (e.g. energy expenditure, aerobic capacity, blood glucose regulation, exercise tolerance, body weight and composition)<br>e. pain<br>3. Improve effects of PT interventions on body structures and functions, by incorporating new scientific discoveries into current interventions, developing new interventions, and/or combining PT interventions with complementary treatments (e.g. medications, regenerative and cellular therapies, brain stimulation)<br>4. Identify activity limitations in children with or at risk for movement-related disorders<br>5. Determine the effects of PT interventions on motor development, motor control and motor learning for postural control, locomotion, upper limb movement, and other motor skills, in children with or at risk for movement related disorders<br>6. Examine relationships between impairments and activity limitations<br>7. Identify participation restrictions in children with or at risk for movement-related disorders

8. Determine the effects of PT interventions on participation in life situations and on quality of life (e.g. school, recreation, domestic life, interpersonal interactions, family relationships, employment)
9. Determine the impact of life transitions (hospital-home, home-school, elementary middle-high school, high school to college/university, school-adult services, and continued access to care/services (i.e., medical home).
10. Examine the impact of PT interventions, including health promotion, on physical activity, sleep patterns, and other developmental outcomes in children who are inactive, overweight or obese due to various factors/diagnoses
11. Examine relationships between impairments, activity limitations, participation, and quality of life in all settings
12. Investigate the effects of technology on the effectiveness of PT interventions, participation, and quality of life (e.g. robotic devices, wearable technologies, interactive gaming systems, virtual reality systems, adaptive exercise equipment, digital health, telehealth, and mobile health).
13. Investigate the effects of assistive mobility devices, orthotics and prosthetics and related novel tools (e.g. 3D printed devices) on gait and other forms of locomotion or developmental skills in children and adults with developmental movement disorders
14. Identify parent, family, home and school characteristics that influence motor development/skill acquisition and responsiveness to PT interventions
15. Identify personal characteristics that influence child development/skill acquisition and responsiveness to PT interventions (e.g. motivation, attention, experience, behavior patterns)

Epidemiology and health services research

1. Investigate factors that influence health policy and health services for children with movement disorders
2. Improve the profession's capacity to conduct child-centered outcomes research by building data infrastructure and by connecting researchers with potential collaborators, mentors, funding agencies, clinicians and consumers.
3. Identify doses of PT interventions that achieve optimal responses (e.g. timing episodes of care, session frequency, duration, intensity, and content, and recommendations for follow through)
4. Evaluate service delivery models for pediatric PT, including school based PT and early intervention, and their effects on child-centered outcomes, family-centered outcomes and cost-effectiveness (e.g. integrative and consultative services, primary-provider model, care coordination, natural environments)
5. Examine differential outcomes as a function of health disparities across diagnoses, ages, races/ethnicities/cultures, and major life transitions
6. Develop effective interventions to address the aforementioned health disparities.
7. Examine the incidence, prevalence and natural course of movement-related health conditions commonly managed by pediatric physical therapists.
8. Evaluate the extent to which pediatric physical therapist decision making is based on available evidence and/or recommended practice guidelines.
9. Evaluate the effects of health promotion efforts by pediatric physical therapists on longitudinal trends in child health and development
10. Identify factors that contribute to utilization and consumer choice in the selection of pediatric physical therapy services

Measurement development and validation

1. Develop and refine measurement tools to identify impairments and monitor changes in the musculoskeletal, neuromuscular and cardiorespiratory systems. (e.g. cardiorespiratory fitness measures for children, or muscle, brain, and other tissue imaging/mapping).
2. Develop and refine pain assessment tools for children
3. Develop and refine measurement tools for prediction of developmental outcomes and responsiveness to intervention based on infant motor behavior
4. Develop and refine measurement tools to identify activity limitations and monitor changes in postural control, locomotion, upper limb movement and other motor skills in children
5. Develop and refine outcome measures specific to various service delivery environments (e.g. school system, early intervention, hospital, NICU)
6. Examine the value of motion sensors and other wearables for treatment planning, including selection of PT interventions, orthotics, and surgical procedures, and effects on child-centered outcomes
7. Develop and refine measures of participation in life situations for children and for adults with developmental disabilities (e.g. fulfillment of life roles in the home, school, community, workplace)
8. Develop and refine measures of quality of life in children
9. Determine minimal detectable changes and minimal clinically important differences for measures used in pediatric physical therapy practice and research
10. Develop a minimum set of measures to evaluate and monitor changes in infants, children, and adults.
11. Develop and refine systems for classifying children with movement-related disorders and determining PT diagnoses

Knowledge translation/implementation science

1. Evaluate the feasibility of a knowledge translation program.
2. Develop a national system for web-based knowledge translation.
3. Examine the efficacy of a mentoring program to improve knowledge translation.
4. Examine the efficacy of knowledge-broker programs to promote knowledge translation in practice.
5. Evaluate specific implementation strategies on knowledge awareness, use and 8 subsequent practice outcomes.

	6. Examine an organization's readiness to implement evidence-based practices and the context specific barriers and facilitators to implementation<br>7. Evaluate how organizations effectively embed new interventions or methods of care into practice.<br>8. Determine the organizational/contextual factors that enable the sustained use of evidence in practice.<br>9. Determine important attributes of clinicians that enhance engagement, knowledge use, and implementation in healthcare settings.<br>**Educational research**<br>1. Conducting or planning for research that investigates a question of importance to the advancement of education in pediatric physical therapy<br>2. Research that advances the knowledge of education and learning processes and the development of the tools and methods necessary to support this endeavor.<br>3. Education Research includes but is not limited to the education of DPT or PhD students, pediatric clinicians, fellows, residents or other learners to better inform pediatric clinical practice<br>4. Area includes multi-institutional studies when possible and when the results of single class or single institution studies have already provided a foundation, studies using established reliable and valid tools for measurement, and studies demonstrating methodological rigor for quantitative, qualitative and mixed designs<br>**Data sharing and analytics**<br>1. Conduct basic, translational, and clinical research through secondary analysis of existing publicly available and accessible national databases and/or administrative records (e.g., electronic medical or health records).<br>2. Formulate new research questions and test new hypotheses using existing individual or combined data sets.<br>3. Examine large datasets to better understand the variations within certain diagnoses/populations and to determine the complex factors affecting future health and movement outcomes.<br>4. Examine records to improve health systems and infrastructure, reduce health inequalities, increase quality of and access to care, and promote health and movement outcomes.<br>5. Create aggregated, harmonized datasets from multiple ongoing studies and/or legacy data from past research studies using common data elements and share data with other researchers for further secondary analysis.<br>6. Apply novel data mining/analytical tools and advanced computational or statistical approaches for large secondary datasets.
VanSwearingen et al., 2022[23]	**Basic science research**<br>1. For genetic, anatomical, biomechanical, physiological, or environmental factors, determine the contribution to or modification of excessive stress, injury, abnormal or accelerated aging of body tissues and systems<br>2. In older adults under conditions of health, injury or disease, examine mechanisms and modifiers of the effects, and optimal dose of physical therapy aging body structural, physiological and functional responses<br>**Clinical research**<br>1. Among older adults in order to guide examination, prevention and treatment of health conditions relevant to physical therapy, define and evaluate relationships among function and disability, health conditions, and personal and environmental factors (eg, International Classification of Functioning, Disability and Health, and applications of such models).<br>2. Examine, define or develop, and evaluate physical therapy interventions and engagement in primary and secondary health promotion, prevention of health decline, and participation for older adults with movement-related health conditions (includes both acute and chronic conditions).<br>3. Among older adults with mobility-assisted ambulation and mobility disability, does a task-oriented walking exercise program improve walking more than a walking endurance exercise program?<br>4. Toward optimization of clinical decision-making in physical therapy for older adults, define and evaluate the efficacy and effectiveness of physical therapist management of clients who are older, based on classification methods, guidelines for criteria or thresholds for progression, decline or indication of function, activity or participation level of ability or independence<br>5. For older adults for whom the margin or tolerance for interventions from a physical, mental, social/emotional or socioeconomic perspective may be limited, determine the effectiveness and efficacy of interventions provided by physical therapists delivered in combination of physical therapy approaches or in combination with other interventions (eg, pharmacological, medical, surgical, or biobehavioral interventions)<br>6. Determine or define, modify and test the effectiveness of varied methods to enhance the ability of older adults to participate and adhere to physical therapy care plans and self-care recommendations, including post-care activity and participation<br>7. Define and determine the effectiveness of various models (ie didactic and clinical education, and technology-driven or web-driven) of professional-level physical therapist education and continuing professional education on clinical management and outcomes, and physical therapy health services delivery for older adults<br>**Epidemiology**<br>1. Examine the incidence, prevalence, and natural course of impairments of body functions and structure, activity limitations, and participation restrictions associated with high-burden health conditions for older adults and vulnerable older adult populations commonly managed by physical therapists<br>2. Investigate the effects of contextual factors (eg, personal and environmental) unique to and common in older adults on the effectiveness of interventions provided by physical therapists

	3. Health services research/policy<br>4. Investigate and improve the performance of the health system for older adults across all settings and addressing delivery models, cost-effectiveness, payment systems, patient access, policy, and research. (Health system and cost effectiveness)<br>5. Investigate and address the impact of older adults' characteristics, values, risk factors, decision-making processes, health literacy, engagement levels on access to, provision of, and outcomes from physical therapy care. (Client centered care)<br>6. Investigate equity in population health and health care for older adults and vulnerable older adult populations, addressing poverty, social determinants of health and well-being, disparities in access to and provision of care, provider bias, and provider cultural competence. (Cultural competence and disparities)<br>7. Investigate the relationship between care processes, mechanisms of care delivery, and physical therapy utilization of older adults and vulnerable older adult populations and physical therapy outcomes.<br>8. Support health services research that targets prevention, diagnosis, treatment, and outcomes for high-burden health conditions of older adults and that is compelling and relevant to policy makers, research funders, and leaders in health care and public health systems. (Policy)<br>**Workforce**<br>1. Examine the effects of staffing patterns on the outcomes of physical therapy for older adults and vulnerable older adult populations; include the identification of factors (eg, use of extenders, mandates) that contribute to variations in productivity<br>2. Investigate factors associated with unmet physical therapy needs of older adults across patient populations, practice settings, and geographic locations; including desirability of practice area, recruitment and retention strategies, and associated health outcomes<br>3. Examine the effects of workforce issues on career pathways for individuals interested in caring for older adults and vulnerable older adulty populations (eg, participation in residency, fellowship, research training)<br>4. Examine the effects of participation in extended clinical training experiences on the development and growth of the workforce prepared to care for older adults and vulnerable older adult populations<br>5. Measurement development and validation<br>6. Develop or refine existing tools and the evidence for use to measure the impact of physical therapy on activity, participation, and quality of life among older adults in acute, long-term residential and community settings. (Particularly combinations of tools / brief batteries to enhance validity<br>7. Develop and provide evidence to guide selection and interpretation of measurement tools for assisted mobility and physical function among older adults. (ie includes mobility with assistive devices, or with the assist of another person)<br>8. Determine and refine standard measurement methods to enhance clinical decision making for older adults; adapt for characteristics of aging body structure and function, age-related health conditions, and specific limitations of activity and participation. To include contemporary technology (eg ultrasound, magnetic and forms of imaging, wearables and the use-ability of these measurement tools given person and environmental factors common among aged
Bowring et al., 2022[24]	1. What is the best type and dose of exercise (physical therapy) for improving muscle strength, flexibility, fitness, balance and function in people with Parkinson's?
Dijkstra et al., 2023[25]	1. What is best practice physical therapy for conditions affecting the young person's hip, focusing primarily on primary cam morphology and its natural history?<br>2. Prognosis after best practice physical therapy and/or arthroscopic hip surgery in different sport/dance/physical activity level cohorts with femoroacetabular impingement syndrome<br>3. Randomised controlled clinical trials to investigate best practice physical therapy vs arthroscopic hip surgery vs sham surgery in cohorts with variable loading demands diagnosed with femoroacetabular impingement syndrome

References

1. Beattie P, Bezner J, Binkley JM, et al. Clinical research agenda for physical therapy. *Physical Therapy.* 2000;80(5):499-+.
2. Soma M, Hosoi T, Yaeda J. Exploring High-Priority Research Questions in Physical Therapy Using the Delphi Study. *Journal of Physical Therapy Science.* 2009;21(4):367-371.
3. Rushton A, Moore A. International identification of research priorities for postgraduate theses in musculoskeletal physical therapy using a modified Delphi technique. *Manual Therapy.* 2010;15(2):142-148.
4. McDonough S, McKenna H, Keeney S, et al. *A Delphi Study to Identify Research Priorities for the Therapy Professions in Northern Ireland-Executive Summary Report.* 2011.

5. Goldstein MS, Scalzitti DA, Craik RL, et al. The revised research agenda for physical therapy. *Physical therapy.* 2011;91(2):165-174.
6. Rankin G, Rushton A, Olver P, Moore A. Chartered Society of Physical therapy's identification of national research priorities for physical therapy using a modified Delphi technique. *Physical therapy.* 2012;98(3):260-272.
7. Pollock A, St George B, Fenton M, Firkins L. Top 10 research priorities relating to life after stroke–consensus from stroke survivors, caregivers, and health professionals. *International journal of Stroke.* 2014;9(3):313-320.
8. Gierisch JM, Myers ER, Schmit KM, et al. Prioritization of patient-centered comparative effectiveness research for osteoarthritis. *Annals of internal medicine.* 2014;160(12):836-841.
9. Boney O, Bell M, Bell N, et al. Identifying research priorities in anaesthesia and perioperative care: final report of the joint National Institute of Academic Anaesthesia/James Lind Alliance Research Priority Setting Partnership. *BMJ Open.* 2015;5(12):e010006.
10. Morris C, Simkiss D, Busk M, et al. Setting research priorities to improve the health of children and young people with neurodisability: a British Academy of Childhood Disability-James Lind Alliance Research Priority Setting Partnership. *BMJ open.* 2015;5(1):e006233.
11. Nast I, Tal A, Schmid S, et al. Physical therapy Research Priorities in Switzerland: Views of the Various Stakeholders. *Physiother Res Int.* 2016;21(3):137-146.
12. Rangan A, Upadhaya S, Regan S, Toye F, Rees JL. Research priorities for shoulder surgery: results of the 2015 James Lind Alliance patient and clinician priority setting partnership. *BMJ open.* 2016;6(4):e010412.
13. KNGF. *Kennis van waarde - onderzoeksagenda fysiotherapie.* 2017.
14. CSP. *Discovering physical therapy research priorities that matter to patients, carers and clinicians.* 2018.
15. Rankin G, Summers R, Cowan K, et al. Identifying Priorities for Physical therapy Research in the UK: the James Lind Alliance Physical therapy Priority Setting Partnership. *Physical therapy.* 2020;107:161-168.
16. Gomes LCS. *Prioridades de Investigação em Fisioterapia músculo-esquelética em Portugal utilizando o método modificado de Delphi*, Instituto Politécnico de Setúbal. Escola Superior de Saúde; 2018.
17. Fernandez MA, Arnel L, Gould J, et al. Research priorities in fragility fractures of the lower limb and pelvis: a UK priority setting partnership with the James Lind Alliance. *BMJ Open.* 2018;8(10):e023301.
18. Wilson T, Martins O, Efrosman M, DiSabatino V, Benbrahim BM, Patterson KK. Physical therapy practice patterns in gait rehabilitation for adults with acquired brain injury. *Brain Inj.* 2019;33(3):333-348.
19. Moerchen VA, Lundeen H, Dole RL. Educational Research Priorities for Pediatric Physical Therapy: A Consensus Study. *Pediatric Physical Therapy.* 2020;32(1):60-69.
20. APTA. *APTA Scientific Research Priorities for the Physical Therapy Profession 2021-2022.* 2021.
21. Pediatrics A. *APTA Academy of Pediatric Physical Therapy (APTA Pediatrics) Research Agenda 2021-2023.* 2021.
22. Bhat AN, Fiss A, O'Neil M, et al. Steps to Revising the APTA Pediatrics Research Agenda. *Pediatr Phys Ther.* 2022;34(3):418-420.
23. VanSwearingen J, Knox S, Lowry KA, et al. Academy of Geriatric Physical Therapy Research Agenda: Rationale for the Development and the Intent for Use. *J Geriatr Phys Ther.* 2022;45(2):76-79.
24. Bowring F, Welch J, Woodward C, et al. Exploration of whether socioeconomic factors affect the results of priority setting partnerships: updating the top 10 research

priorities for the management of Parkinson's in an international setting. *BMJ Open.* 2022;12(6):e049530.
25. Dijkstra HP, Mc Auliffe S, Ardern CL, et al. Oxford consensus on primary cam morphology and femoroacetabular impingement syndrome: part 2-research priorities on conditions affecting the young person's hip. *Br J Sports Med.* 2022;57(6):342-358

S3. Research questions within every research priority

Table S3. Global research agenda (top 9 research priorities) retrieved from the content analysis.

Research priority	Research questions from documents
1. Establish the (cost)effectiveness of different physical therapy interventions	• What is the effectiveness of segmental mobilization/manipulation in reducing impairment and improving functional outcomes in patients with reduced segmental mobility?<br>• Are manual techniques effective in the treatment of impairments and functional limitations?<br>• What is the effect of exercise (duration, intensity, and type) on bone density?<br>• Can physical therapy interventions for patients with spasticity or rigidity improve function?<br>• What interventions designed to change movement strategies can be used for patients with lumbar segmental instability, and what is the optimal pattern?<br>• What interventions designed to decrease pain and paresthesias can be used for patients with upper-extremity entrapment syndromes, and what is the optimal pattern?<br>• Does immediate postoperative physical therapy intervention improve the rate of recovery of function in patients with impaired cardiovascular function, and, if so, how?<br>• Does immediate postoperative physical therapy intervention affect the rate of recovery of function in patients following orthopedic surgery, and, if so, how?<br>• Can interval training be used to improve physiological and functional outcomes in frail elderly people? If yes, can the process of interval training be standardized with frail elderly people?<br>• Are outcomes of treatment following peripheral nerve injury using neuromuscular re-education improved by early assessment and staged interventions?<br>• What is the relative effectiveness of immobilization versus mobilization in patients with musculoskeletal impairments on tissue healing and recovery of function?<br>• What are the interactions between physical therapy interventions and pharmacological interventions?<br>• Is the difference between natural recovery and the effect of the treatment clear?<br>• Can an individual medical expense and people's medical expenses reduce doing physical therapy?<br>• What balance training is of possible practical use for elderly?

	• Is physical therapy effective for higher brain dysfunction?<br>• What is the effect of the therapeutic exercise in cerebral palsy?<br>• What are the effects of stretching?<br>• What are the effects of physical activity and exercise on sub populations with musculoskeletal disorders?<br>• What are the clinical guidelines for the assessment and management of shoulder, knee and ankle problems?<br>• An examination of the role of exercise in improving mental health of mild/moderate depression<br>• Cost benefit analysis of the provision of services<br>• More effective incorporation of health economics within future research design<br>• To research the benefits of physical therapy intervention in promoting an enablement ethos with chronic conditions<br>• Research designed to assess the impact of physical activity on health and wellbeing<br>• The effectiveness of exercise interventions in lymphoedema management<br>• Assessing the effectiveness of treatments in the management of chronic pain including exercise, acupuncture, education, hypnosis and biopsychosocial approaches<br>• An assessment of the effectiveness of interventions in the management of back pain including traction, manual therapy and core stability strategies<br>• Contrasting the clinical effectiveness of the use of classes with one-to-one treatment approaches<br>• An investigation into the benefits of exercise based rehabilitation of soft tissue injury<br>• Evaluation of the role of exercise in cancer rehabilitation – intensify/frequency etc.<br>• The impact of exercise intensity on symptom management and recovery in long term conditions<br>• The effectiveness of individualised development care for preterm infants born at less than 32 weeks gestation<br>• Examine the effects of physical therapy interventions that are provided independently or in combination on cellular structural properties and physiological responses of healthy, injured, or diseased body tissues.<br>• Define the role for physical therapy in the maturation and modelling of genetically engineered tissues.<br>• Develop new physical therapy interventions to promote tissue growth and adaptation.

	• Examine the impact of health promotion interventions that include the involvement of physical therapists on activity and participation of individuals with movement disorders.<br>• Evaluate or develop effective interventions to prevent or reduce the risk of disability associated with common health conditions.<br>• Determine the effects of interventions provided by physical therapists to address secondary prevention in patients/clients with chronic diseases (eg, diabetes, obesity, arthritis, neurological, other disorders).<br>• Determine the effectiveness and efficacy of interventions provided by physical therapists across relevant domains of health.<br>• Determine the effectiveness and efficacy of interventions provided by physical therapists delivered in combination with other interventions (eg, medical, surgical, or biobehavioral interventions).<br>• Develop and test the effectiveness of physical therapist interventions for primary and secondary conditions or disability.<br>• Develop and test the effectiveness of physical therapist interventions to optimize treatment outcomes for specific subgroups of patients/clients<br>• Develop and test the effectiveness of methods to improve patient/client adherence to the plan of care and self-management.<br>• Perform economic evaluation of specific physical therapy interventions.<br>• Evaluate the effect of physical therapy service delivery models on economic and patient/client outcomes and consumer choice.<br>• Evaluate the comparative cost and/or cost-effectiveness of specific physical therapy interventions compared with or in combination with other interventions.<br>• Evaluate the effectiveness of shared clinical decision-making schemes between the patient/client and therapist on clinical outcomes and costs<br>• Assess the impact of continuity of physical therapy services on outcomes.<br>• Exploration of interventions/strategies to increase patients' adherence to/concordance with exercise programmes (adherence to exercise programmes)<br>• Exercise as medicine: prescription of exercise (i.e. type, dosage, environment) for patients with chronic, long-term musculoskeletal conditions (exercise prescription)

	• The clinical and cost effectiveness of physical therapy for patients with patellofemoral pain (effectiveness of physical therapy management: lower body quadrant problems)<br>• Development and evaluation of physical activity programmes for older people (effectiveness of physical therapy management: specific conditions)<br>• The role of physical therapists in promoting exercise and physical activity – preventative healthcare, not only treatment (exercise prescription)<br>• Effectiveness of graduated rehabilitation for patients with shoulder pain (effectiveness of physical therapy management: upper body quadrant problems)<br>• What are the benefits of physical therapy rehabilitation for patients with low back pain? (effectiveness of physical therapy management: low back pain)<br>• What is the impact and value of physical therapy on return to work for people with musculoskeletal conditions? (return to work)<br>• How do we improve the outcomes for people with shoulder impingement syndrome (effectiveness of physical therapy management: upper body quadrant problems)<br>• Management of anterior knee pain in children and in adolescents (effectiveness of physical therapy management: paediatric conditions)<br>• What is the optimum exercise intervention for individuals with osteoarthritis of the knee? (effectiveness of physical therapy management: osteoarthritis)<br>• Do workplace modifications and physical therapy treatments facilitate early return to work, with cost benefits to the employer? (return to work)<br>• Early physical therapy for acute low back pain in a working population (effectiveness of physical therapy management: low back pain<br>• Effectiveness of early physical therapy intervention on anterior knee pain in children and young people (effectiveness of physical therapy management: paediatric conditions)<br>• The clinical and cost effectiveness of physical therapy for anterior cruciate ligament injuries (effectiveness of physical therapy management: lower body quadrant problems)<br>• Comparing physical therapist-led vs exercise professional-led exercise prescription (exercise prescription)

	• Effectiveness of rehabilitation compared to surgery for anterior cruciate ligament rupture (effectiveness of physical therapy management: lower body quadrant problems)<br>• What is the role, content and effectiveness of self-management strategies in long term neurological conditions? (effectiveness of specific interventions: self-management)<br>• What are the benefits of stroke rehabilitation on function and quality of life in the longer term (>6 months and >1 year post stroke)? (effectiveness of physical therapy management: post stroke)<br>• Is self-practice of repetitive, high intensity, task-specific activities beneficial to the motor recovery of patients with stroke? (effectiveness of physical therapy management: post stroke)<br>• Specific therapy interventions in the community setting for the continuing support of people with long term neurological conditions (effectiveness of physical therapy management: other neurological conditions)<br>• What are the benefits of aerobic exercise for people with long term neurological conditions, and what are effective service delivery models? (exercise/physical activity)<br>• Effectiveness of exercise and fitness programmes for stroke survivors, including the long-term benefits on function, quality of life and subsequent stroke prevention (exercise/physical activity)<br>• The efficacy of therapy interventions for patients early after stroke onset (effectiveness of physical therapy management: post stroke)<br>• Cost effectiveness of service delivery models to increase the intensity of therapy in stroke units (service provision: post stroke)<br>• Demonstrating the value of physical therapy interventions in maintaining mobility for patients with Multiple Sclerosis (MS) (effectiveness of physical therapy management: multiple sclerosis)<br>• What role should physical therapy play in the management of people with long-term neurological conditions? (effectiveness of specific interventions)<br>• Stroke rehabilitation for residents of care homes (effectiveness of physical therapy management: post stroke<br>• Investigation of the efficacy of physical therapy for patients with different types of ataxia (effectiveness of physical therapy management: ataxia)<br>• Promotion of physical fitness and activity for children and young adults with Cerebral Palsy and

	Neurodisability (effectiveness of physical therapy management: paediatric neurological conditions)<br>• Contracture management: how should physical therapists treat and advise patients with neurological conditions to prevent or reduce contracture development that impedes functional recovery? (effectiveness of specific interventions: postural)<br>• What is the impact and cost-effectiveness of post-stroke rehabilitation for the long term stroke survivor? (parameters of intervention)<br>• Establishing an effective at-home exercise programme, focusing on improving balance, for patients with progressive ataxia (effectiveness of physical therapy management: ataxia)<br>• What role do physical interventions play in combination with botulinum toxin (BTX) in the management of spasticity in patients following central neurological damage? (effectiveness of specific interventions)<br>• Access to physical activity and fitness activities for people with long term neurological conditions (exercise/physical activity)<br>• The effectiveness of modern therapy adjuncts e.g. functional electrical stimulation, Seabo Flex, constraint induced therapy (effectiveness of specific interventions: adjuncts/equipment/assistive technologies)<br>• The recovery profile of patients with spinal cord injury (SCI) and the influence of early mobilisation (effectiveness of physical therapy management: spinal cord injury)<br>• Efficacy of cardiovascular fitness and energy expenditure markers in the initial rehabilitation and in the long term management of patients with spinal cord injury (SCI) (effectiveness of physical therapy management: spinal cord injury)<br>• Improving adherence of patients with Multiple Sclerosis (MS) in remaining physically active following a physical therapy intervention (effectiveness of physical therapy management: multiple sclerosis)<br>• Effectiveness of self-management programmes (SMPs) for patients with stroke (effectiveness of specific interventions: self management)<br>• Can physical therapy reduce falls in patients with Parkinsons Disease? (effectiveness of physical therapy management: other neurological conditions)<br>• Exploring the best strategies for motor relearning in patients with cognitive or language deficits

	(effectiveness of physical therapy management: other neurological conditions)<br>• Self-management in cerebral palsy: at the time of transition to adult services, would education in self management of physical problems associated with cerebral palsy improve the quality of life and socio-economic independence of young adults with cerebral palsy? (transition from paediatric to adult services)<br>• The effect of intermittent rehabilitation over a long-term period on patients' function and participation in society (parameters of intervention)<br>• How can physical therapists help to improve adherence to physical activity programmes developed for patients with neurological conditions in the community? (exercise/physical activity)<br>• The effects and experiences of physical activity interventions for non-ambulatory patients with stroke or other long-term neurological conditions (exercise/physical activity)<br>• Effectiveness of long-term stroke support services for stroke survivors and their carers (service provision: post stroke)<br>• An evaluation of exercises and exercise devices for patients with progressive ataxia using wheelchairs (effectiveness of physical therapy management: ataxia)<br>• Effectiveness of gait rehabilitation for patients with stroke (effectiveness of physical therapy management: post stroke)<br>• Demonstrating the value of physical therapy for patients with Multiple Sclerosis (MS) following a relapse (effectiveness of physical therapy management: multiple sclerosis)<br>• Falls management in people with long term neurological conditions, especially with stroke and Multiple Sclerosis (guidelines/policy: development, implementation and impact)<br>• What are the benefits, cost-effectiveness and long term impact on patient outcomes of delivering 7-day physical therapy services within secondary care? (service provision: 7-day working)<br>• The effect of a physical therapist-led early mobility programme in Intensive Therapy Units (ITUs) on patients' long-term outcomes of function, mobility and quality of life (effectiveness of physical therapy management: critically ill patients)<br>• Long term benefits and cost effectiveness of different models of physical therapy intervention for new lower limb amputees (effectiveness of physical therapy management: amputees)

	• Comparative work on the role of the physical therapist in post-critical care rehabilitation and follow-up clinics, to look at long-term outcomes and possible predictors of functional outcome (effectiveness of physical therapy management: critically ill patients)<br>• Investigating interventions which could enhance recovery in patients with critical illness (effectiveness of physical therapy management: critically ill patients)<br>• Pulmonary rehabilitation intervention in patients with early stage Chronic Obstructive Pulmonary Disease (COPD) (effectiveness of cardiopulmonary rehabilitation)<br>• The efficacy of the cough assist technique in patients with neuromuscular disease (effectiveness of specific interventions: airway clearance)<br>• Exercise interventions for patients with critical illness: feasibility and physiological and functional outcomes (effectiveness of specific interventions: exercise/physical activity)<br>• Rehabilitation programmes in palliative care (effectiveness of specific interventions)<br>• Short- and long-term effectiveness of pulmonary rehabilitation (PR) initiated either during or post admission for an exacerbation of Chronic Obstructive Pulmonary Disease (COPD) (effectiveness of cardiopulmonary rehabilitation)<br>• The effects of early mobilisation and rehabilitation for paediatric patients in the Intensive Care Unit (ICU) (effectiveness of physical therapy management: critically ill patients)<br>• Manual chest physical therapy techniques for secretion clearance for patients who are intubated and mechanically ventilated (effectiveness of physical therapy management: critically ill patients<br>• Improving adherence to chronic disease management services such as pulmonary rehabilitation and exercise programmes for patients with chronic lung disease (effectiveness of specific interventions: exercise/physical activity)<br>• What is the efficacy of self-management and educational interventions for patients with respiratory compromise? (adherence)<br>• Effectiveness of physical therapist-led exercise classes for people with long term conditions (tailored treatment)<br>• Benefits of physical activity and exercise in adult patients with congenital heart disease (the role of the physical therapist)

	• The role of physical therapy in the care of patients with dementia to assist in the maintenance of functional independence (effectiveness of specific interventions: airway clearance)<br>• Investigating the physiological mechanisms of airway clearance interventions, evaluating different techniques, and short and long term outcomes in patients with non-cystic-fibrosis bronchiectasis (effectiveness of specific interventions: exercise/physical activity)<br>• Comparing the effectiveness of different models of pulmonary rehabilitation (effectiveness of cardiopulmonary rehabilitation)<br>• Cost effectiveness and long-term benefits of physical therapy management of patients with acute exacerbations of Chronic Obstructive Pulmonary Disease (COPD) (service provision)<br>• Long term effectiveness of physical therapy for patients with cystic fibrosis (effectiveness of physical therapy management: cystic fibrosis)<br>• Efficacy of exercise training for low functioning and high-risk patients (effectiveness of specific interventions: exercise/physical activity)<br>• The effectiveness of physical therapy service provision for young men with Duchenne's muscular dystrophy at the point of transition from paediatric to adult services (service provision<br>• Effectiveness of programmes to change physical activity behaviour for people with long term conditions (physical activity)<br>• Developing effective collaborations with third sector exercise/activity providers for people with chronic musculoskeletal pain (physical therapy role in exercise intervention)<br>• Optimum levels of exercise in the treatment and prevention of mental health problems, for example, depression and dementia (effectiveness of exercise: managing depression)<br>• Evidence to support the physical therapist's role in delivering exercise programmes in a variety of settings (physical therapy role in exercise intervention)<br>• Physical therapy management of chronic musculoskeletal dysfunction in the older patient (effectiveness of physical therapy)<br>• Promoting engagement in physical activity for people with long-term conditions (physical activity)<br>• Physical therapy interventions for people with dementia in acute hospital settings (effectiveness of physical therapy management of dementia)

	- Effectiveness of physical exercise in the recovery of patients with chronic obstructive pulmonary disease (COPD) after acute exacerbations (effectiveness of exercise intervention) - How can physical therapists educate and influence patients in the need to engage more regularly in physical activity? (physical activity) - Physical therapy and cognitive behavioural therapy (CBT) (Effectiveness of physical therapy) - Effectiveness of early supported discharge in getting patients out of hospital (service provision) - Optimising the self-management support given by physical therapists to patients with chronic diseases (health behaviour change) - Physical therapy management of patients with specific conditions who also have mental health problems (education/continuing professional development) - Effective tools for assessing and treating pain in patients with dementia (effectiveness of physical therapy management of dementia) - The role of non-specific exercise in management of long term conditions (effectiveness of exercise intervention) - The role of physical therapy in promoting return to work for people with musculoskeletal and mental health conditions (role of the physical therapist in public health/health promotion) - Effectiveness of exercise and relaxation interventions for patients with common mental health conditions. (effectiveness of exercise: managing depression) - Long term effectiveness of providing community rehabilitation services for patients with long term conditions or those recovering from serious illness. (community based physical therapy services) - Does exercise decrease recurrence and improve survival in patients with cancer? (effectiveness of exercise: patients with cancer) - Optimising the frequency and intensity of exercise for older patients. (effectiveness of exercise: older people) - How effective are physical therapists in influencing patients to be more active? (physical activity) - What is the best physical therapy regime for recovery after stroke? - What are the comparative safety and effectiveness of usual care nonsurgical therapies (pharmacotherapy, injections, physical therapy/exercise, and weight loss) or combinations of usual care nonsurgical therapies to prevent

	progression of and disability from OA? Are these effects maintained (i.e., long-term outcomes) over time?<br>• How can preoperative exercise or fitness training, including physical therapy, improve outcomes after surgery?<br>• Are any types of physical therapy (eg, Bobath, Neuro-Developmental Therapy, hydro, constraint, strength-training, etc) more or less effective to promote motor functioning in children and young people with neurodisability (eg, cerebral palsy, acquired brain injury)?<br>• Research should develop and evaluate efficient physical therapy treatment methods<br>• Research should develop and evaluate programmes in the field of secondary prevention (e.g. activity promotion and prevention of falls in elderly people and prevention of chronic manifestations of disease)<br>• Research should develop and evaluate programmes in the field of primary prevention (e.g. movement related health promotion in schools and prevention of back pain<br>• Does early mobilisation and physical therapy after shoulder surgery improve patient outcome compared to standard immobilisation and physical therapy?<br>• Are patients (including older age groups) with rotator cuff tendon tears in their shoulder best treated with surgery or physical therapy<br>• What is the effectiveness of (early) physical therapy on (non-)medical cost-savings and/or substitution of more expensive forms of health care (like surgery or expensive medication)?<br>• What is the effectiveness of physical therapy on physical functioning, compared to usual care or no intervention (wait-and-see-policy)?<br>• What is the effectiveness of physical therapy on work absence, return to work and work-related (societal) costs?<br>• What is the added value of behavioral interventions within the physical therapy treatment on patient adherence and sustained treatment effects, and what are the required competences of physical therapists to provide this?<br>• When health problems are developing, at what point is physical therapy most/least effective for improving patient results compared to no physical therapy? What factors affect this?<br>• When used by physical therapists, what methods are effective in helping patients to make health

	changes, engage with treatment, check their progress, or manage their health after discharge<br>• To stop health problems occurring or worsening, what physical therapy treatments, advice or approaches are safe and effective? Where more than one treatment/approach works, which work best and in what dose?<br>• What parts of physical therapy treatments cause behaviour change or physical improvement?<br>• When trying to improve patient and service outcomes, what types of exercises, doses and methods of delivery are effective?<br>• Cost effectiveness of various physical therapy modalities, physical therapy vs other interventions and different response models<br>• Effectiveness of physical therapy in musculoskeletal symptoms and conditions<br>• Effectiveness of interventions/strategies to promote adherence to the intervention and self-management of the musculoskeletal condition<br>• What is the best physical therapy and/or occupational therapy regime for adults during their in-hospital recovery from a fragility fracture of the lower limb?<br>• What is the best physical therapy and/or occupational therapy regime for adults during out-of-hospital recovery from a fragility fracture of the lower limb?<br>• What are the best physical therapies to treat adults with a fear of falling after a lower limb fragility fracture?<br>• Development and validation of acquired brain injury-specific gait treatments<br>• Interventions: Investigate the impact of physical therapy on population outcomes (health and wellness) across the lifespan; including total cost of care, quality of life, reductions in disability, and reduction in the burden of care<br>• Telehealth: Determine the effectiveness of telehealth delivery, clinical examination via telehealth, and patient engagement strategies, and identify patient populations most likely to benefit from telehealth<br>• Value: Determine value (cost-effectiveness, impact on longer-term clinical outcomes, and quality of life) of physical therapy compared with non-rehabilitation treatments for clinical conditions appropriate for physical therapy<br>• Treatment: Determine the effects of physical therapist interventions in addressing secondary prevention and health promotion in individuals

	with chronic diseases (diabetes, obesity, arthritis, neurological, and other disorders)<br>• Delivery models: Explore cost analysis and assess outcomes of delivery of care models (one-on-one versus multiple patients per provider versus team based) and facility ownership (outpatient corporate versus outpatient independently owned versus outpatient hospital-affiliated versus inpatient hospital based) and payment models (insurance versus cash pay)<br>• Utilization and cost: Explore the financial benefits for physical therapy. Compare utilization and costs (outcomes/costs) of the numerous practice areas for physical therapists (identifying where physical therapists add value to the system, where physical therapists add additional cost, where physical therapy services add redundancy, etc.)<br>• Payment and insurance: Explore the impact of various payment models and insurance providers on patient and clinical outcomes and downstream costs and utilization<br>• Determine the effects of PT interventions and other factors such as nutrition on:<br>o skeletal muscle and tendon (e.g. development and modification of muscle architecture, strength, power, muscle and tendon length, activation patterns, and recovery from injury or surgery)<br>o bones and joints (e.g. development and modification of bone density and architecture, infant head shape, alignment of joints, and recovery from injury or surgery)<br>o central and peripheral nervous system (e.g. development and modification of neural pathways and networks, activity-dependent neural adaptation, regeneration, restoration and compensatory changes after nervous system damage)<br>o the cardiorespiratory system, metabolism and caloric balance (e.g. energy expenditure, aerobic capacity, blood glucose regulation, exercise tolerance, body weight and composition)<br>o pain<br>• Improve effects of PT interventions on body structures and functions, by incorporating new scientific discoveries into current interventions,

	developing new interventions, and/or combining PT interventions with complementary treatments (e.g. medications, regenerative and cellular therapies, brain stimulation<br>• Determine the effects of PT interventions on motor development, motor control and motor learning for postural control, locomotion, upper limb movement, and other motor skills, in children with or at risk for movement related disorders<br>• Determine the effects of PT interventions on participation in life situations and on quality of life (e.g. school, recreation, domestic life, interpersonal interactions, family relationships, employment)<br>• Examine the impact of PT interventions, including health promotion, on physical activity, sleep patterns, and other developmental outcomes in children who are inactive, overweight or obese due to various factors/diagnoses<br>• Investigate the effects of assistive mobility devices, orthotics and prosthetics and related novel tools (e.g. 3D printed devices) on gait and other forms of locomotion or developmental skills in children and adults with developmental movement disorders<br>• Evaluate service delivery models for pediatric PT, including school based PT and early intervention, and their effects on child-centered outcomes, family-centered outcomes and cost-effectiveness (e.g. integrative and consultative services, primary-provider model, care coordination, natural environments)<br>• Examine differential outcomes as a function of health disparities across diagnoses, ages, races/ethnicities/cultures, and major life transitions<br>• Develop effective interventions to address the aforementioned health disparities<br>• Evaluate the effects of health promotion efforts by pediatric physical therapists on longitudinal trends in child health and development<br>• Examine, define or develop, and evaluate physical therapy interventions and engagement in primary and secondary health promotion, prevention of health decline, and participation for older adults with movement-related health conditions (includes both acute and chronic conditions).<br>• Among older adults with mobility-assisted ambulation and mobility disability, does a task-oriented walking exercise program improve walking more than a walking endurance exercise program?<br>• Toward optimization of clinical decision-making in physical therapy for older adults, define and evaluate the efficacy and effectiveness of physical

	therapist management of clients who are older, based on classification methods, guidelines for criteria or thresholds for progression, decline or indication of function, activity or participation level of ability or independence<br>• For older adults for whom the margin or tolerance for interventions from a physical, mental, social/emotional or socioeconomic perspective may be limited, determine the effectiveness and efficacy of interventions provided by physical therapists delivered in combination of physical therapy approaches or in combination with other interventions (eg, pharmacological, medical, surgical, or biobehavioral interventions)<br>• Determine or define, modify and test the effectiveness of varied methods to enhance the ability of older adults to participate and adhere to physical therapy care plans and self-care recommendations, including post-care activity and participation<br>• What is best practice physical therapy for conditions affecting the young person's hip, focusing primarily on primary cam morphology and its natural history?<br>• Randomised controlled clinical trials to investigate best practice physical therapy vs arthroscopic hip surgery vs sham surgery in cohorts with variable loading demands diagnosed with femoroacetabular impingement syndrome<br>• What role does physical therapy play in keeping people with specific conditions out of hospital, for example, those with respiratory conditions, mobility problems or at risk of falling? (physical therapy in public health)<br>• Effectiveness of physical therapy in the prevention of pain/injury/musculoskeletal conditions<br>• What are the influences of education on patients with acute/chronic low back pain?
2. Research the optimal service delivery models, structures and processes of physical therapy interventions	• When health problems are developing, at what point is physical therapy most/least effective for improving patient results compared to no physical therapy? What factors affect this?<br>• What types of exercises, doses and methods of delivery are effective in stopping health problems occurring or worsening?<br>• What is the best type and dose of exercise (physical therapy) for improving muscle strength, flexibility, fitness, balance and function in people with Parkinson's?

	• What are the conditions of repetition and practice (whole/part, intermittent/continuous, attended/unattended, number of trials per day) that optimize function in people with neuromuscular dysfunction?<br>• Are there optimal time periods for interventions that influence pathology, impairment, functional limitation, and disability in patients in whom multiple episodes of care are expected over the life span?<br>• What is the effect of various intensities and durations of intervention on the rate and degree of functional recovery after anterior cruciate ligament injury?<br>• What is the optimal dose/response relationship for interventions (eg, aerobic and strengthening exercise, manual therapy, physical agents, traction/mechanical modalities, flexibility), given a specific category of a classification system for low back pain?<br>• Does the coordination of exercise and surgical interventions affect patient outcomes, and, if so, what is the optimal pattern of intervention?<br>• Does the coordination of exercise and pharmacological interventions affect patient outcomes, and, if so, what is the optimal pattern of intervention<br>• What is the optimal resource schedule and utilization to achieve a desired effect or outcome for a given diagnosis?<br>• How have changes resulting from health care reorganization affected the quality of physical therapy services, access to physical therapy services, patient satisfaction, staff productivity, staff longevity, and professional development?<br>• How does the requirement of referral before treatment affect whether patients have access to and are likely to utilize physical therapy services?<br>• What are the factors that determine whether patients have access to and are likely to utilize physical therapy services?<br>• For how long and for what sort of patient should the physical therapy be done?<br>• What is the impact of evidence within OMT?<br>• What strategies work in helping to integrate evidence into scientific practice?<br>• An exploration of the impact of the pressure of targets, waiting lists and the volume of repeat referrals on achieving intervention outcomes that reflect the needs and expectations of patients

	• Identification of optimal duration and intensity of treatment and engagement with patients linked to outcomes<br>• Identification of areas for development in the structure of how therapy is provided – self-management in adults, parent-led therapy in children, and group therapy versus one-to-one approaches<br>• An exploration of optimal assessment and treatment times for physical therapy appointments – do longer appointment times result in better long-term outcomes?<br>• Determine the optimal dose of physical therapy interventions (frequency, duration, intensity) to achieve optimal cellular and physiological adaptation/response of body tissues and systems.<br>• Determine the physical therapist's role and impact in contemporary delivery models on prevention of diseases and their secondary side effects.<br>• Determine interactions among interventions provided by physical therapists.<br>• Determine the effects of frequency, duration, intensity, and timing of interventions provided by the physical therapist.<br>• Determine the relationship between documentation and payment.<br>• Determine disparities in the access to and provision of physical therapy and their impact on outcomes.<br>• Describe patterns of physical therapy use and identify factors that contribute to variation in utilization.<br>• Examine the effects of staffing patterns on the outcomes of physical therapy.<br>• Assess productivity of physical therapists in various settings and identify factors (eg, use of extenders, mandates) that contribute to variations in productivity.<br>• Identify and test the best methods to assess past, current, and future demand and unmet needs for physical therapy.<br>• Identify the demand for services among populations underserved by physical therapists<br>• Investigate the relationship between the distribution of physical therapists and population health outcomes.<br>• Effectiveness of self-referral to physical therapy for musculoskeletal conditions (service provision: self-referral to physical therapy)<br>• What is best practice in the rehabilitation of the upper limb in patients with stroke with respect to

	timing, content and dosage? (effectiveness of physical therapy management: post stroke)<br>• Dose response studies – how do intensity, frequency and duration of physical therapy interventions relate to outcomes for different stages of stroke recovery and for other neurological conditions? (parameters of intervention)<br>• The development of appropriate service delivery models for use in long term (service provision: post stroke)<br>• Implementing intensive repetitive practice for patients with stroke (service provision: post stroke)<br>• What is the optimal frequency and timing of physical therapy interventions for children with cerebral palsy? (effectiveness of physical therapy management: paediatric neurological conditions)<br>• What are the components and necessary timing of effective complex postural management for patients with severe neurological impairment, such as late stage multiple sclerosis or severe traumatic brain injury? (effectiveness of specific interventions: postural)<br>• The optimum level of physical therapy for children and young people with neurological conditions (parameters of intervention)<br>• Does access to an emergency on-call physical therapy service improve patient outcomes? (service provision)<br>• What are the main modalities of treatment for patients with phantom pain after amputation in the UK? (effectiveness of physical therapy management: pain after amputation)<br>• The frequency, intensity and timing of exercise required to optimise rehabilitation for patients with critical illness (effectiveness of specific interventions: exercise/physical activity)<br>• Exploring access to appropriate physical therapy and rehabilitation for patients with dementia disorders in the UK, to maintain/improve mental and physical health and wellbeing and independence (effectiveness of physical therapy management of dementia)<br>• How do we integrate models of working with fitness instructors, physical therapists and GPs to get the best outcomes for patients? (physical therapy role in exercise intervention<br>• The role of physical therapy in 'fit note' schemes (role of the physical therapist in public health/health promotion)

	• Optimum staffing ratios and reasonable waiting times in relation to effective service delivery (service provision)<br>• Does the timing and intensity of 'early' intervention (eg, providing information, physical therapy, speech and language therapy, occupational therapy, etc) alter effectiveness of therapies for infants and young children with neurodisability, including those without specific diagnosis? What is the appropriate age of onset/strategies/dosage/direction of therapy interventions?<br>• Research should develop and evaluate pilot projects for direct access<br>• Research should focus on evaluating the current system in terms of the future quality in physical therapy practice<br>• Research should develop multidisciplinary health care networks addressing changing societal needs<br>• Research should evaluate the effects of physical therapy networks<br>• Which physical therapy interventions that have been proven to be effective need to be (better) implemented, and/or which physical therapy interventions that have been proven to be ineffective need to be (better) de-implemented in daily practice?<br>• Which adaptations in physical therapy interventions are necessary to be effective in complex patient groups, like patients with multi-morbidity, intellectual disability or frailty?<br>• What is the optimal content, intensity and duration of physical therapy interventions and for which specific patients ('personalized care') or specific subgroups ('stratified care') to optimize the size of effects?<br>• How does waiting for physical therapy affect patient and service outcomes?<br>• How can access to physical therapy be improved for groups who have reduced access?<br>• How does the amount of physical therapy received affect results for patients and services? What are optimal session lengths, frequency and duration of treatment?<br>• What do the people who fund services and internal budget holders understand about the role of physical therapy and how do they make funding decisions?<br>• What's the availability of physical therapy services nationally, how does this compare between specialisms, countries, or to documented need? What affects service availability across the UK?

	• Do staffing levels and skill mix impact patient and service outcomes? What are the best staffing levels and skill mixes in different areas of physical therapy and how do these compare to current staffing provision?<br>• What are patients offered nationally in terms of treatment sessions, appointment times and follow-on care? How is it checked that this is enough?<br>• How are different physical therapy services provided, staffed and accessed across the UK and what influences this?<br>• Definition of parameters/dose to optimize effects of interventions<br>• Determine the impact of life transitions (hospital-home, home-school, elementary middle-high school, high school to college/university, school-adult services), and continued access to care/services (i.e., medical home).<br>• Identify doses of PT interventions that achieve optimal responses (e.g., timing episodes of care, session frequency, duration, intensity, and content, and recommendations for follow through)<br>• Identify factors that contribute to utilization and consumer choice in the selection of pediatric physical therapy services<br>• Examine an organization's readiness to implement evidence-based practices and the context specific barriers and facilitators to implementation<br>• Evaluate how organizations effectively embed new interventions or methods of care into practice.<br>• Determine the organizational/contextual factors that enable the sustained use of evidence in practice.<br>• Determine important attributes of clinicians that enhance engagement, knowledge use, and implementation in healthcare settings<br>• Examine records to improve health systems and infrastructure, reduce health inequalities, increase quality of and access to care, and promote health and movement outcomes.<br>• Define and determine the effectiveness of various models (ie didactic and clinical education, and technology-driven or web-driven) of professional-level physical therapist education and continuing professional education on clinical management and outcomes, and physical therapy health services delivery for older adults<br>• Investigate and improve the performance of the health system for older adults across all settings and addressing delivery models, cost-effectiveness,

	payment systems, patient access, policy, and research. (Health system and cost effectiveness)<br>• Investigate and address the impact of older adults' characteristics, values, risk factors, decision-making processes, health literacy, engagement levels on access to, provision of, and outcomes from physical therapy care. (Client centered care)<br>• Investigate equity in population health and health care for older adults and vulnerable older adult populations, addressing poverty, social determinants of health and well-being, disparities in access to and provision of care, provider bias, and provider cultural competence. (Cultural competence and disparities)<br>• Investigate the relationship between care processes, mechanisms of care delivery, and physical therapy utilization of older adults and vulnerable older adult populations and physical therapy outcomes.<br>• Support health services research that targets prevention, diagnosis, treatment, and outcomes for high-burden health conditions of older adults and that is compelling and relevant to policy makers, research funders, and leaders in health care and public health systems. (Policy)<br>• Examine the effects of staffing patterns on the outcomes of physical therapy for older adults and vulnerable older adult populations; include the identification of factors (eg, use of extenders, mandates) that contribute to variations in productivity<br>• New ways of delivering falls prevention therapy for older people (service provision) What are the factors that affect cost for physical therapy services within specific diagnostic groups?<br>• Is there a difference in patient outcomes and costs dependent on whether services for a given diagnostic condition are provided by physical therapists or others?<br>• What is the effect of the availability, cost, and payment source of physical therapy services on patient outcomes?<br>• Exploring the best strategies for motor relearning in patients with cognitive or language deficits (effectiveness of physical therapy management: other neurological conditions)<br>• What methods do physical therapists use to treat patients, to help them gain skills to manage their condition and to use them in their daily lives?

	• Investigate factors that influence health policy and health services for children with movement disorders
3. Explore the best models of physical therapy education, and professional development and quality	• Is the physical therapy practiced based on evidence?<br>• Is the form of the clinical practice an ideal way in a physical therapy education?<br>• What approaches in education assist in the development of clinical reasoning skills?<br>• What is the efficacy of teaching OMT techniques?<br>• What are the existing models of postgraduate education in OMT?<br>• Determine the best methods to foster career development and leadership in physical therapy.<br>• What is the effectiveness of training to enhance the validity of palpation skills?<br>• Determine the optimal criteria for board certification.<br>• Evaluate the effect of clinical education models on clinical outcomes, passing rates on the National Physical Therapy Examination, and employment settings after graduation.<br>• Determine the impact of professional-level physical therapist education on professional behaviors.<br>• Assess the effectiveness of models of professional education on clinical performance.<br>• Determine the relationship between student cultural competency and clinical decision making.<br>• Evaluate the effectiveness of different methods used to improve cultural competence.<br>• Develop and evaluate the most effective methods for facilitating physical therapist acquisition and use of available information resources for evidence-based practice.<br>• Evaluate the skills needed by practitioners to provide optimal patient/client care, patient/client advocacy, and cost-effective care<br>• Establish the extent to which physical therapists deliver services in accordance with recommended guidelines for specific conditions and its impact on outcomes.<br>• Examine the cultural competence of physical therapists and physical therapist assistants and its impact on intervention.<br>• Investigate the influence of health policies on practice patterns and outcomes.<br>• Evaluate methods to enhance adherence to recommended practice guidelines.

	• Determine factors that contribute to the attractiveness of practicing in various settings and geographic regions.<br>• Determine factors that contribute to the retention of physical therapists across various settings and geographic regions.<br>• Determine the effectiveness of recruitment and retention initiatives in reducing the gap between supply and demand in various practice settings.<br>• Identify variables that influence the decision of whether or not to enter the physical therapy profession.<br>• Assess the impact of expanded scope of practice on supply and demand.<br>• Examine the effects of workforce issues on career pathways (eg, participation in residency, fellowship, research training).<br>• Examine the effects of participation in extended clinical training experiences on workforce<br>• What training and development is needed by physical therapists at pre- and post-registration to ensure they can be effective in facilitating health behaviour change? (education/continuing professional development)<br>• Do physical therapists have the skills to advise patients on exercising for health? (physical therapy role in exercise intervention)<br>• Research should further develop and evaluate educational systems tailored best on physical therapy professional education<br>• Physical therapy should invest in educational research and in the further development and evaluation of curricula<br>• Research should explore changing requirements of the physical therapy profession (e.g. due to a higher share of the population of older people or increasing amount of chronic diseases)<br>• Research should explore effects of the shift of the professional education to the level of University of Applied Sciences<br>• Research should focus on necessary competencies of physical therapists required for the implementation of direct access<br>• Research should contribute to the further development of physical therapy continuing education<br>• What training is available to physical therapists for developing their skills either working with different conditions or using more specialist approaches?

	• Utilisation of evidence-based practice (barriers, adherence, results from implementation strategies)<br>• Identify the relevant pediatric physical therapy content (knowledge, skills, abilities, experiences, behaviors, attitudes, etc) that should be included in pediatric physical therapy education<br>• Identify effective methods of measuring outcomes of educational experiences related to pediatric physical therapy that demonstrate that students or practitioners have acquired the identified relevant knowledge, skills, abilities, experiences, behaviors, attitudes, etc.<br>• Identify effective teaching and learning methods/strategies for the instruction, practice, and evaluation of skills, behaviors, and attitudes that are meaningful to pediatric physical therapy outcomes (including but not limited to experiential learning, clinical reasoning, simulation, computer assisted)<br>• Identify indicators of readiness for pediatric physical therapy practice<br>• Identify the preferred amount of experiential learning that is meaningful to pediatric physical therapy education outcomes<br>• Identify the effect (on curriculum, outcomes, student learning/readiness for practice, etc) of the published Essential Competencies in Entry-Level Pediatric Physical Therapy Education<br>• Identify definitions and indicators for excellence in pediatric physical therapy education<br>• Identify the expectations of employer/employee readiness for pediatric physical therapy practice and what effect these expectations have on curricula<br>• Identify the benefits and challenges of differences in curricular content (depth and breadth) on pediatric physical therapy education outcomes<br>• Determine the effectiveness of strategies/methods/practices for promoting and enhancing knowledge translation, continued competency, and advanced practice in pediatric physical therapy<br>• Identify the benefits and challenges of different curricular models on pediatric physical therapy education outcomes<br>• Determine the effectiveness of current strategies/methods/practices for preparing individuals as educators, including both academic and clinical, for pediatric physical therapy education

	• Improve the profession's capacity to conduct child-centered outcomes research by building data infrastructure and by connecting researchers with potential collaborators, mentors, funding agencies, clinicians and consumers.<br>• Evaluate the extent to which pediatric physical therapist decision making is based on available evidence and/or recommended practice guidelines.<br>• Evaluate the feasibility of a knowledge translation program.<br>• Develop a national system for web-based knowledge translation.<br>• Examine the efficacy of a mentoring program to improve knowledge translation.<br>• Examine the efficacy of knowledge-broker programs to promote knowledge translation in practice.<br>• Evaluate specific implementation strategies on knowledge awareness, use and 8 subsequent practice outcomes.<br>• Conducting or planning for research that investigates a question of importance to the advancement of education in pediatric physical therapy<br>• Research that advances the knowledge of education and learning processes and the development of the tools and methods necessary to support this endeavor.<br>• Education Research includes but is not limited to the education of DPT or PhD students, pediatric clinicians, fellows, residents or other learners to better inform pediatric clinical practice<br>• Area includes multi-institutional studies when possible and when the results of single class or single institution studies have already provided a foundation, studies using established reliable and valid tools for measurement, and studies demonstrating methodological rigor for quantitative, qualitative and mixed designs<br>• Examine the effects of workforce issues on career pathways for individuals interested in caring for older adults and vulnerable older adulty populations (eg, participation in residency, fellowship, research training)<br>• Examine the effects of participation in extended clinical training experiences on the development and growth of the workforce prepared to care for older adults and vulnerable older adult populations<br>• An exploration of the relationship between skill mix and clinical outcomes

	• Evaluate the effect of physical therapist post professional specialty training on clinical decision making and patient/client outcomes .
4. Development and study measurement instruments relevant to physical therapy	• What are the relationships between self-report of function and observed measures?<br>• What are the psychometric properties of performance-based and self-assessment measures of physical function designed to predict functional limitations and disability in elderly people?<br>• What is the reliability of segmental mobility testing in the cervical spine?<br>• What are the reliability and validity of assessment of pronation of the foot in patients with knee pain?<br>• What is the reliability of the McKenzie classification system for the cervical spine?<br>• What is the normal response to a range of examination tests?<br>• What is the intra-rater reliability of a broad range of assessment tools?<br>• What is the inter-rater reliability of a broad range of assessment tools?<br>• What is the accuracy of a broad range of assessment tools?<br>• What is the face validity of a broad range of assessment tools?<br>• What is the content validity of a broad range of assessment tools?<br>• What is the concurrent validity of a broad range of assessment tools?<br>• What is the sensitivity of a broad range of assessment tools?<br>• What is the specificity of a broad range of assessment tools?<br>• What is the validity of new clinical prediction rules for treatment outcome?<br>• What is the predictive value of a broad range of assessment tools?<br>• How is patient satisfaction evaluated?<br>• What is the clinical and linguistic validation of existing questionnaires?<br>• What is the clinical utilisation of various outcome measures?<br>• What performance-based outcome measures are most appropriate for mechanical neck disorders?<br>• What is an appropriate functional testing outcome measure for use in LBP?<br>• An investigation into how exercise capabilities should be assessed dependent on disease state including the identification of an exercise prescription

	• Research into the use of functional tests in assessment<br>• Identify thresholds for adequate physical function to optimize outcomes and prevent injury.<br>• Develop or adapt measures of effectiveness and impact of physical therapy at the community level.<br>• Develop new tools or refine existing tools to measure the impact of physical therapy on activity, participation, and quality of life.<br>• Provide evidence to guide selection and interpretation of measurement tools for specific purposes, conditions, and populations.<br>• Develop and test a minimum set of measures to evaluate the process and clinical outcomes for specific conditions and populations.<br>• Develop reliable and valid measures of cultural competence of physical therapy providers and students.<br>• Determine how contemporary technology (eg, ultrasound, gene array, magnetic resonance) can be used to measure the effects of injury/disease and physical therapy intervention on body structure and function.<br>• Determine optimal measurement methods to enhance clinical decision making for specific conditions and populations<br>• Harnessing the potential of routine use of Patient Reported Outcome Measures in physical therapy practice (outcomes of care)<br>• Developing the right tools to assess outcomes of physical therapy interventions (outcomes of care)<br>• Developing and testing a brief screening tool that can assist physical therapists to explore obstacles to return to work (return to work)<br>• Developing outcome measures to evaluate the effectiveness of physical therapy for patients in palliative care (outcome measures)<br>• Effective tools for assessing and treating pain in patients with dementia (effectiveness of physical therapy management of dementia)<br>• What are valid outcome measures in evaluating physical therapy practice and patient benefit? (outcomes of care)<br>• Which core outcome sets of patient-relevant and crucial (generic if possible) outcome measures and minimally clinically relevant improvements should be used by physical therapists in daily practice?<br>• How is patient progress and/or the results of physical therapy treatment measured? How is service performance measured and checked?

	• Development and validation of gait-specific outcome measures<br>• Development and validation of technology for treatment of gait impairments<br>• Develop and refine measurement tools to identify impairments and monitor changes in the musculoskeletal, neuromuscular and cardiorespiratory systems. (e.g. cardiorespiratory fitness measures for children, or muscle, brain, and other tissue imaging/mapping).<br>• Develop and refine pain assessment tools for children<br>• Develop and refine measurement tools for prediction of developmental outcomes and responsiveness to intervention based on infant motor behavior<br>• Develop and refine measurement tools to identify activity limitations and monitor changes in postural control, locomotion, upper limb movement and other motor skills in children<br>• Develop and refine outcome measures specific to various service delivery environments (e.g. school system, early intervention, hospital, NICU)<br>• Develop and refine measures of participation in life situations for children and for adults with developmental disabilities (e.g. fulfillment of life roles in the home, school, community, workplace)<br>• Develop and refine measures of quality of life in children<br>• Determine minimal detectable changes and minimal clinically important differences for measures used in pediatric physical therapy practice and research<br>• Develop a minimum set of measures to evaluate and monitor changes in infants, children, and adults.<br>• Measurement development and validation<br>• Develop or refine existing tools and the evidence for use to measure the impact of physical therapy on activity, participation, and quality of life among older adults in acute, long-term residential and community settings. (Particularly combinations of tools / brief batteries to enhance validity<br>• Develop and provide evidence to guide selection and interpretation of measurement tools for assisted mobility and physical function among older adults. (ie includes mobility with assistive devices, or with the assist of another person)<br>• Determine and refine standard measurement methods to enhance clinical decision making for older adults; adapt for characteristics of aging body

	structure and function, age-related health conditions, and specific limitations of activity and participation. To include contemporary technology (eg ultrasound, magnetic and forms of imaging, wearables) and the use-ability of these measurement tools given person and environmental factors common among aged<br>• Are there measures that can be used to predict independent function in an urban community, and, if so, what measures and at what thresholds?<br>• Are there measures of ambulation that can be used to predict independent function in various communities, and, if so, at what thresholds?
5. Conduct research to better understand mechanisms behind disability, physical therapy treatments and patient classification systems	• What are commonly performed physical functional tasks, and how do they differ across the life span?<br>• What are the physical findings in patients with vertigo?<br>• Is there a relationship between weight-bearing exercises and the risk of fractures for people with bone demineralization, and, if so, what is the relationship between exercise and risk?<br>• How do impairments affect disability in patients?<br>• To what extent do variables such as pharmacology, psychosocial factors, and environmental factors influence the relationship among impairment, functional limitation, and disability in people receiving physical therapy interventions?<br>• What are the modifiable risk factors for cumulative trauma syndrome?<br>• Identify impairments in children with or at risk for movement-related disorders - Explore factors associated with movement-related impairments<br>• Identify activity limitations in children with or at risk for movement-related disorders<br>• Identify participation restrictions in children with or at risk for movement-related disorders<br>• Do motor control strategies differ in people with low back pain compared with people without low back pain, and, if so, how?<br>• What is the method of motion learning?<br>• What are the relationships between lifestyle, the quantity of activities of daily living, and physical fitness?<br>• Do physical therapists' knowledge, attitude, culture, understanding, and expectations affect the outcome of physical therapy interventions, and, if so, how?<br>• What are the descriptive patterns of musculoskeletal disorders?

	• Identify how genetic, anatomical, biomechanical, physiological, or environmental factors contribute to excessive stress, injury, or abnormal development of body tissues and systems.<br>• Determine if modifiable genetic, anatomical, biomechanical, physiological, or environmental factors can decrease risk of excessive stress, injury, or abnormal development of body tissues and systems.<br>• Investigate the factors that modify the response to physical therapy intervention and positive tissue adaptation (eg, genetic, functional, structural, psychosocial, and physiological factors).<br>• Examine skill acquisition and motor development in individuals with movement disorders.<br>• Examine the relationship between biomarkers and impairments in body structure and function, limitations in activity, and restrictions in participation<br>• Determine the mechanisms by which physical therapy interventions modify disease and age-related or injury-induced changes in normal cellular structure and function using appropriate human and animal models.<br>• Examine the incidence, prevalence, and natural course of health conditions (disorders, diseases, and injuries) commonly managed by physical therapists.<br>• Examine the incidence, prevalence, and natural course of impairments of body functions and structure, activity limitations, and participation restrictions associated with health conditions commonly managed by physical therapists.<br>• Exploring barriers to behaviour change in the management of long-term conditions (health behaviour change)<br>• What are the physiological effects of different physical therapy treatments?<br>• Mechanisms that justify the effects of physical therapy interventions (manual therapy, exercise)<br>• Physiological effects (nervous system; physiological markers) of physical therapy interventions (manual therapy, education, exercise)<br>• Explore the mechanisms of tissue damage and repair in the musculoskeletal, neuromuscular and cardiorespiratory systems<br>• Describe development in infants and children that are typically developing, at risk for movement-related disorders, or are diagnosed with movement-related disorders

	• Investigate critical/sensitive periods for neuroplasticity and motor development (e.g. in infancy or after neural injury)<br>• Examine relationships between motor development and other domains of child development (e.g. cognitive, social, emotional, and language)<br>• Examine brain-behavior relationships during functional behaviors<br>• Explore physical activity/participation levels in children and adults<br>• Examine relationships between impairments and activity limitations<br>• Examine relationships between impairments, activity limitations, participation, and quality of life in all settings<br>• Examine the incidence, prevalence and natural course of movement-related health conditions commonly managed by pediatric physical therapists.<br>• For genetic, anatomical, biomechanical, physiological, or environmental factors, determine the contribution to or modification of excessive stress, injury, abnormal or accelerated aging of body tissues and systems<br>• In older adults under conditions of health, injury or disease, examine mechanisms and modifiers of the effects, and optimal dose of physical therapy aging body structural, physiological and functional responses<br>• Examine the incidence, prevalence, and natural course of impairments of body functions and structure, activity limitations, and participation restrictions associated with high-burden health conditions for older adults and vulnerable older adult populations commonly managed by physical therapists<br>• Understanding the impact of gait dysfunction on participation in daily life<br>• Understanding the impact of gait dysfunction on community integration<br>• Understanding impairments that contribute to gait dysfunction<br>• Investigating the physiological mechanisms of airway clearance interventions, evaluating different techniques, and short and long term outcomes in patients with non-cystic-fibrosis bronchiectasis (effectiveness of specific interventions: exercise/physical activity)

	• What factors can be used to classify patients with thoracic disorders?<br>• What factors can be used to classify patients following a cerebrovascular accident?<br>• When multiple tests and measures are used, how is the information weighted in determining a diagnosis?<br>• What are the criteria for diagnosis of different presentations?<br>• What are the common physical/subjective/bio-psychosocial characteristics of patient sub groups eg; acute low back pain, chronic low back pain, whiplash associated disorder, tennis elbow, OA hip etc?<br>• Determine the relationships among levels of functioning and disability, health conditions, and contextual factors for conditions commonly managed by physical therapists (eg, International Classification of Functioning, Disability and Health).<br>• Develop and evaluate effective patient/client classification methods to optimize clinical decision making for physical therapist management of patients/clients.<br>• Research should develop physical therapy assessment and diagnosis further, as specification of patients' problems is a prerequisite for tailored, cost-effective treatment<br>• Research should focus on physical therapy assessment and diagnosis in the area of chronic, highly prevalent diseases (e.g. obesity and chronic back pain)<br>• Develop and refine systems for classifying children with movement-related disorders and determining PT diagnoses
6. Explore patients' needs, expectations, experience and contextual factors and how they influence treatment outcomes	• Do patient knowledge, attitude, culture, understanding, and expectations affect the outcome of physical therapy interventions, and, if so, how?<br>• Identify contextual factors (eg, personal and environmental) that affect prognosis.<br>• How does waiting for physical therapy affect patient and service outcomes?<br>• What are the factors that motivate patients to adhere to a plan of care?<br>• How does the physical environment in which the patient must function (eg, work requirements, mobility barriers) influence the effectiveness of treatment interventions?<br>• How does the environment in which the patient must function influence the choice of physical therapy interventions?

	• Are there changes to behavior and the environment that can be used to enhance function and prevent impairments, and, if so, what is the optimal pattern of use to achieve a therapeutic outcome?<br>• Do changes to behavior and the environment reduce the incidence of work-related cumulative trauma disorder?<br>• Do payer source and policies influence satisfaction with access to physical therapy services in patients with acute conditions?<br>• Do payer source and policies influence satisfaction with access to physical therapy services in patients with chronic conditions?<br>• Is the patient satisfied with the provided physical therapy (content or result)?<br>• What is the patient's experience of OMT?<br>• What factors contribute to patient satisfaction?<br>• What are patient expectations of OMT service delivery?<br>• What factors determine patient satisfaction with OMT?<br>• What are the pain experiences of patients with acute/chronic low back pain?<br>• What are the patient's experiences of the treatment of chronic pain?<br>• What is the influence of patient expectations on OMT treatment and outcomes?<br>• Investigate the effects of contextual factors (eg, personal and environmental) on the effectiveness of interventions provided by physical therapists.<br>• Examine the interaction among access, culture, and health literacy on physical therapy outcomes.<br>• Motivating the demotivated to exercise (adherence to exercise programmes)<br>• What are the service needs of patients with end stage respiratory disease? (patient centred practice)<br>• Developing a better understanding of the reality of how amputees wear and use their limb in the normal day (effectiveness of physical therapy management: amputees)<br>• Research should address barriers and facilitators within physical therapist–patient interaction<br>• Research should engage in experiences and behaviours of patients (e.g. motivation, compliance, coping and psychosocial problems)<br>• What are the best ways to deliver physical therapy services to meet patients' needs and improve outcomes for patients and services?<br>• What are patients' expectations regarding recovery, how do these compare to physical therapists' views

	and, where recovery is not possible, how is this managed?<br>• What approaches are effective for enabling parents, relations or carers to support physical therapy treatment or to help patients to manage their own health problem?<br>• How well do patients recall physical therapy advice and to what extent do patients follow this advice?<br>• What do patients expect of physical therapy and understand in terms of remaining healthy, their condition and their role in self-management?<br>• Patient satisfaction with recommended physical therapy/interventions<br>• Among older adults in order to guide examination, prevention and treatment of health conditions relevant to physical therapy, define and evaluate relationships among function and disability, health conditions, and personal and environmental factors (eg, International Classification of Functioning, Disability and Health, and applications of such models).<br>• How can patient characteristics and environmental factors be used to predict adherence to home programs?<br>• Is there a relationship between a patient's satisfaction with care and adherence to his or her physical therapy care plan?<br>• Disparities: Investigate health equity in rehabilitation (disparities across race and ethnicities, various age groups, cultures, and socioeconomic status) — and its impact on access to care and outcomes, reducing clinician biases, and institutional and community level oppression<br>• Social determinants of health: Investigate the mechanisms and mediators by which upstream social determinants of health (food insecurity, physical environment, access-to-care, education, employment/working conditions, transportation barriers, early child development, etc.) shape the development of disparities in impairments, activity limitations, participation restrictions, and poor rehabilitation outcomes, in order to identify targets for multilevel intervention<br>• Identify parent, family, home and school characteristics that influence motor development/skill acquisition and responsiveness to PT interventions

	• Identify personal characteristics that influence child development/skill acquisition and responsiveness to PT interventions (e.g. motivation, attention, experience, behavior patterns)<br>• Investigate the effects of contextual factors (eg, personal and environmental) unique to and common in older adults on the effectiveness of interventions provided by physical therapists<br>• Investigate factors associated with unmet physical therapy needs of older adults across patient populations, practice settings, and geographic locations; including desirability of practice area, recruitment and retention strategies, and associated health outcomes<br>• What are the characteristics of the sub group populations responding or not responding to OMT?
7. Search for prognostic outcomes and investigate responses to physical therapy	• What factors influence the transfer of functional skills from the therapeutic environment to the community?<br>• What tests and measures should be used to predict the physical therapy services patients will require upon discharge from inpatient care to achieve maximum function?<br>• Do measures of impairment and function predict a person's ability to work or return to work?<br>• What impairment-level and functional-level measures predict work capacities?<br>• What are the variables, if any, that predict return of function in individuals following stroke?<br>• Are there elements of motor control and cognitive function that can be used to predict physical function in individuals with central nervous system dysfunction?<br>• How are responses to exercise different in patients with neurological impairments?<br>• Are there critical levels and elements of motor control that must be present to permit household ambulation in individuals with brain dysfunction, and, if so, what are they?<br>• What are the characteristics of people who respond to various forms of therapy for low back pain?<br>• What factors appear to predict outcome of care in individual subgroups?<br>• What are the quality of life issues affecting treatment outcome?<br>• An exploration of the factors associated with adherence to exercise and physical fitness programmes

	- Identify factors that predict the risks of, or protection from, health conditions (injury, disorders, and disease). - Determine predictors of recovery from adverse effects associated with medical or surgical treatment. - The role of physical therapy in preventing the next episode of spinal pain; and to what extent does adherence influence the outcome of physical therapy management regimes (public health) - What information from measures can be used to predict physical function in community-dwelling elderly people? - What measurements of ambulation are useful for predicting patients' function? - Are there measures that can be used to predict independent function in an urban community, and, if so, what measures and at what thresholds? - Are there measures of ambulation that can be used to predict independent function in various communities, and, if so, at what thresholds? - Are there measurements from the initial examination that predict future or concurrent mobility or disability, and, if so, how? - Potential prognostic indicators for therapeutic interventions for patients with stroke (tailored treatment) - Research should evaluate the influence of treatment type, frequency, intensity and length on socioeconomic and patient relevant outcomes (e.g. improvement of autonomy in older age, reduction of work absence and improvement of quality of life) - What factors predict the onset of health problems, patient responses to physical therapy or their abilities to make health changes/self-manage? Which patients (if any) are likely to benefit most/least from physical therapy? - Identification and assessment of risk factors for musculoskeletal injuries/conditions - Explore factors that affect growth and development of muscles, bones, neural networks, and other tissues and systems that contribute to movement - Prognosis after best practice physical therapy and/or arthroscopic hip surgery in different sport/dance/physical activity level cohorts with femoroacetabular impingement syndrome - Does the use of risk prediction models to determine which patients receive post-operative physical therapy (effectiveness of specific interventions: exercise/physical activity)

8. Explore and establish clinical decision-making strategies/tools	• What information from the diagnosis/prognosis is used in patient/client management?<br>• What factors beyond the diagnosis/prognosis determine patient/client management?<br>• What combination of examination data can be used to guide clinical decision making for patients with pain in the sacroiliac region?<br>• How does information from the systems review influence tests and measures chosen?<br>• How does information from the history influence tests and measures chosen?<br>• Do measures of postural alignment in people with spinal disorders influence clinical decision making, and, if so, how?<br>• What factors are used by physical therapists to determine their recommendations of settings to which patients are discharged?<br>• What are the necessary criteria for motion independence, activities of daily living and motor function?<br>• What is the relevance and use of red flags in the management of musculoskeletal disorders?<br>• What is the relevance and use of yellow flags in the management of musculoskeletal disorders?<br>• Develop and evaluate models of health and disability to guide the investigation, prevention, and treatment of health conditions relevant to physical therapy.<br>• Develop and test the effectiveness of decision support tools to facilitate evidence-based physical therapist decision making.<br>• Evaluate the effect of physical therapist post professional specialty training on clinical decision making and patient/client outcomes.<br>• Investigate factors that influence patient/client choices when selecting a health care provider or making treatment decisions.<br>• Develop and evaluate new methods for incorporating patient/client values and expectations into the decision-making process.<br>• Evaluate the effectiveness of shared clinical decision-making schemes between the patient/client and therapist on clinical outcomes and costs.<br>• Develop innovative medical informatics applications for physical therapy and assess their impact on clinical decision making.<br>• Which criteria (generic if possible) to start or to end a treatment of physical therapy should be used by physical therapists in daily practice?

	• How do physical therapists decide on what their treatment plans include and/or when to refer on? What influences the types of evidence they use?<br>• Investigate and address the impact of older adults' characteristics, values, risk factors, decision-making processes, health literacy, engagement levels on access to, provision of, and outcomes from physical therapy care. (Client centered care)<br>• What are the clinical reasoning processes used in OMT?
9. Investigate the added value of technology and big data for physical therapy	• Which, if any, devices and equipment (assistive, adaptive, orthotic, protective, supportive, or prosthetic) can be used by physical therapists to enhance function and prevent impairments, and what is the pattern of use to achieve a therapeutic outcome?<br>• What research is needed for creating a database for effective general physical therapy?<br>• Identify technologies to assist physical therapists in developing prevention approaches that optimize outcome.<br>• The role of assistive technologies in the restoration of motor function in people with neurological conditions (effectiveness of specific interventions: adjuncts/equipment/assistive technologies)<br>• Research should improve knowledge of new technologies and their possible impact on physical therapy (e.g. new surgical techniques, assistive devices and orthoses)<br>• Research should contribute to the implementation of new technologies for physical therapy (e.g electronic patient file and virtual reality in technology for diagnosis and treatment of movement disorders)<br>• Telehealth: Determine the effectiveness of telehealth delivery, clinical examination via telehealth, and patient engagement strategies, and identify patient populations most likely to benefit from telehealth<br>• What is the feasibility and added value of technological devices for physical therapists, aiming at optimizing the diagnostic or therapeutic process?<br>• What is the feasibility and added value of 'internet-based care' or 'blended care', aiming at enhancing patient adherence and sustained treatment effects, compared to completely supervised physical therapy, usual care or no intervention ('wait-and-see-policy')?<br>• What is the possible role of 'big data', collected through technological devices, in monitoring health (reductions) and physical functioning in specific

	patient groups, or in identifying diseases in an early phase in health people?<br>• Investigate the effects of technology on the effectiveness of PT interventions, participation, and quality of life (e.g. robotic devices, wearable technologies, interactive gaming systems, virtual reality systems, adaptive exercise equipment, digital health, telehealth, and mobile health).<br>• Examine the value of motion sensors and other wearables for treatment planning, including selection of PT interventions, orthotics, and surgical procedures, and effects on child-centered outcomes<br>• Create aggregated, harmonized datasets from multiple ongoing studies and/or legacy data from past research studies using common data elements and share data with other researchers for further secondary analysis.<br>• Apply novel data mining/analytical tools and advanced computational or statistical approaches for large secondary datasets.<br>• Determine and refine standard measurement methods to enhance clinical decision making for older adults; adapt for characteristics of aging body structure and function, age-related health conditions, and specific limitations of activity and participation. To include contemporary technology (eg ultrasound, magnetic and forms of imaging, wearables) and the use-ability of these measurement tools given person and environmental factors common among aged<br>• Examine large datasets to better understand the variations within certain diagnoses/populations and to determine the complex factors affecting future health and movement outcomes.<br>• Conduct basic, translational, and clinical research through secondary analysis of existing publicly available and accessible national databases and/or administrative records (e.g., electronic medical or health records).<br>• Formulate new research questions and test new hypotheses using existing individual or combined data sets.<br>• Develop innovative medical informatics applications for physical therapy and assess their impact on clinical decision making.<br>• Identify technologies to assist physical therapists in determining patient/client classification.
